# Specific phytochemicals in floral nectar up‐regulate genes involved in longevity regulation and xenobiotic metabolism, extending mosquito life span

**DOI:** 10.1002/ece3.7665

**Published:** 2021-05-25

**Authors:** Teresia M. Njoroge, Bernarda Calla, May R. Berenbaum, Christopher M. Stone

**Affiliations:** ^1^ Department of Entomology University of Illinois at Urbana‐Champaign Urbana IL USA; ^2^ Illinois Natural History Survey University of Illinois at Urbana‐Champaign Champaign IL USA

**Keywords:** gene expression, longevity, mosquito, nectar phytochemicals, sugar‐feeding behavior

## Abstract

During nectar feeding, mosquitoes ingest a plethora of phytochemicals present in nectar. The ecological and physiological impacts of these ingested phytochemicals on the disease vectors are poorly understood. In this study, we evaluated the effects of three nectar phytochemicals‐‐ caffeine, *p*‐coumaric acid, and quercetin‐‐on longevity, fecundity, and sugar‐feeding behavior of the Asian tiger mosquito (*Aedes albopictus)*. Adult females of *Ae*. *albopictus* were provided continuous access to 10% sucrose supplemented with one of the three phytochemicals and their fecundity, longevity, and the amount of sucrose consumed determined. Transcriptome response of *Ae*. *albopictus* females to *p*‐coumaric acid and quercetin was also evaluated. Dietary quercetin and *p*‐coumaric acid enhanced the longevity of female *Ae*. *albopictus*, while caffeine resulted in reduced sugar consumption and enhanced fecundity of gravid females. RNA‐seq analyses identified 237 genes that were differentially expressed (DE) in mosquitoes consuming *p*‐coumaric acid or quercetin relative to mosquitoes consuming an unamended sucrose solution diet. Among the DE genes, several encoding antioxidant enzymes, cytochrome P450s, and heat shock proteins were upregulated, whereas histones were downregulated. Overall, our findings show that consuming certain nectar phytochemicals can enhance adult longevity of female Asian tiger mosquitoes, apparently by differentially regulating the expression level of genes involved in longevity and xenobiotic metabolism; this has potential impacts not only on life span but also on vectorial capacity and insecticide resistance.

## Graphical abstract

The study investigated the effects of nectar phytochemicals caffeine, *p*‐coumaric acid, and quercetin on longevity, fecundity, and sugar‐feeding behavior of female *Aedes albopictus*. Additionally, we did a whole‐transcriptome analysis of female *Ae*. *albopictus* consuming sucrose diets supplemented with *p*‐coumaric acid or quercetin to characterize their transcriptional profile. Quercetin and *p*‐coumaric acid significantly enhanced the longevity of the mosquitoes while caffeine consumption resulted in reduced sugar feeding and enhanced fecundity. Dietary *p*‐coumaric acid or quercetin led to a change in expression of genes associated with longevity regulation (superoxidase dismutase, heat shock proteins, and histones) and xenobiotic metabolism (P450s and other enzymes involved in degradation of toxins).

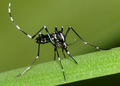

## 1 INTRODUCTION

Flowering plants secrete both floral and extra‐floral nectars, which play important ecological roles as attractants and food rewards for mutualists. Floral nectar is produced inside the flower while extra‐floral nectar is secreted by other vegetative parts of the plant and is commonly involved in indirect defense against herbivores (Nepi et al., 2018). Floral nectar is the principal reward for animals in exchange for pollination services (Ollerton et al., 2011). Insects, primarily hymenopterans, lepidopterans, and dipterans, are major plant pollinators and hence key beneficiaries of plant nectar as a food resource (Ollerton et al., 2011; Peach & Gries, 2020). Mosquitoes are widely assumed to be nectar thieves, and, when compared to other insects, they may be less effective in pollen transfer, but there is abundant evidence that they are important and even essential pollinators for a diversity of flowering plants (Peach & Gries, 2016, 2020).

In addition to nutrients such as sugars, amino acids, and vitamins that are required by nectar feeders for growth and development, floral nectar contains secondary metabolites (phytochemicals), albeit in smaller concentrations than in foliage and other plant tissues. These phytochemicals include phenolics, terpenoids, coumarins, and alkaloids, among many other structural types (Adler, 2000; Nicolson & Thornburg, 2007). Although phytochemicals function primarily in defense against herbivores and microorganisms (Bennett & Wallsgrove, 1994), nectar feeders experience both beneficial and detrimental effects from these compounds. Because phytochemicals vary geographically and are taxonomically idiosyncratically distributed, nectar chemistry from the perspective of pollinating insects varies in time and space (Nicolson & Thornburg, 2007).

While nectar primary metabolites such as sugars, amino acids, and proteins are important sources of energy and nitrogen and influence the physiological, immune, nutritional, and behavioral responses of a variety of nectar‐feeders (Alm et al., 1990; Foster, 1995; Mevi‐Schütz & Erhardt, 2005; Rivera‐Pérez et al., 2017; Nepi et al., 2018; Nicolson & Thornburg, 2007), less is known about the effects of nectar phytochemicals on these insects. Studies evaluating the ecological significance of nectar phytochemicals on nectar‐feeding insects have focused on the western honey bee (*Apis mellifera*) and bumble bees (*Bombus* spp.) due to their economic importance as managed plant pollinators. The alkaloids anabasine, nicotine, and caffeine supplemented in diets affect feeding preferences and enhance learning and memory in adult honey and bumble bees (Adler, 2000; Adler & Irwin, 2005; Baracchi et al., 2017; Si et al., 2005; Singaravelan et al., 2005; Wright et al., 2013), whereas phenolic acids and flavonols influence flower visitor feeding behavior, suppress pathogens and parasites, enhance longevity, and increase pesticide tolerance by upregulating genes involved in xenobiotic metabolism, immunity, and caste determination (Baracchi et al., 2015; Bernklau et al., 2019; Hagler & Buchmann, 1993; Liao et al., 2017a, 2017b; Lin Liu et al., 2004; Liu et al., 2007; Mao et al., 2013, 2015; Palmer‐Young et al., 2017; Richardson et al., 2015; Singaravelan et al., 2005).

For most species of mosquitoes, nectar is an essential dietary requirement for adults of both sexes (Foster, 1995). Male mosquitoes feed solely on plant sugars while females of many mosquito species also require a blood meal to complete the gonotrophic cycle. An energy‐rich diet from nectar is known to influence mosquito survival, fecundity, host‐seeking behavior, blood feeding, and capacity to transmit pathogens (Foster, 1995; Stone & Foster, 2013). Floral and extra‐floral nectars and honeydew comprise the main sources of sugar meals for mosquitoes, although other sources can include fruit juices, plant sap, plant exudates, or even foliage (Foster, 1995; Gary & Foster, 2004; Peach & Gries, 2020). The choice of nectar sources by nectar feeders including mosquitoes is likely determined through a combination of the nutritional quality of the nectar, the visual and olfactory attractiveness, and accessibility of the host plant (Manda et al., 2007; Müller et al., 2011; Nicolson & Thornburg, 2007; Nikbakhtzadeh et al., 2014; Nyasembe et al., 2012).

Studies exploring mosquito–nectar interactions have focused mainly on *Anopheles* mosquitoes and highlighted the ecological significance of nectar from different plant species. Different nectar sources are known to influence mosquito survival, fecundity, host‐seeking behavior, biting rate (Gary & Foster, 2001, 2004; Impoinvil et al., 2004; Manda et al., 2007; Nikbakhtzadeh et al., 2016), and vectorial capacity (Stone et al., 2012; Ebrahimi et al., 2018), as well as the infection rate and intensity of *Plasmodium falciparum* in *Anopheles gambiae,* an observation that led to a suggestion that the mechanism underlying these differences may be due to variation in the phytochemical content of the nectar (Hien et al., 2016).

A growing number of studies have examined the impacts of secondary metabolites on mosquitoes (Johnson & Riehle, 2015; Nunes et al., 2016; Nyasembe et al., 2015). For instance, sucrose diets containing the polyphenols genistein, resveratrol, and quercetin extended the adult life span and reduced the proliferation of gut microbiota in female *Ae. aegypti* (Nunes et al., 2016). However, a comprehensive understanding of the impacts of nectar phytochemicals on various aspects of mosquito life‐history traits, including, sugar‐feeding behavior, vector competence, metabolism, and immunity, has remained elusive.

**FIGURE 1:**
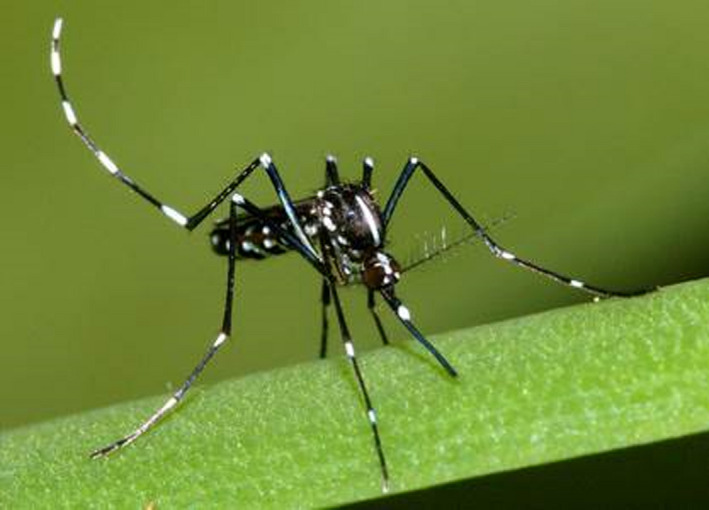
Asian tiger mosquito, *Aedes albopictus*, resting on a leaf. Photo courtesy of Susan Ellis (www.bugwood.org)

In this study, we used the Asian tiger mosquito *Ae*. *albopictus* (Figure 1) to conduct laboratory assays aimed at determining the impacts of nectar phytochemicals on mosquito behavior and physiology. *Aedes albopictus* is an invasive species with high ecological plasticity that has become established in temperate regions of Europe and America (Swanson et al., 2000; Johnson et al., 2017). This container‐breeding species inhabits peri‐urban and rural areas and is a vector of epidemiologically important human arboviruses, including dengue and chikungunya, and is also capable of transmitting a wide array of viruses under laboratory conditions (Paupy et al., 2009). Specifically, we examined the effects of the alkaloid caffeine, the phenolic acid *p*‐coumaric acid, and the flavonol quercetin on longevity, fecundity, and sugar‐feeding behavior of the mosquitoes. Additionally, using next‐generation sequencing, we undertook a whole‐transcriptome analysis of female *Ae*. *albopictus* consuming sucrose diets supplemented with p‐coumaric acid or quercetin to characterize their transcriptional profile. The specific phytochemicals were selected because they are found in nectar, honey, and pollen of many plant species and have been demonstrated to influence the sugar‐feeding behavior and to enhance memory (caffeine) of adult honey bees and bumble bees (Singaravelan et al., 2005; Wright et al., 2013), in addition to extending the life span (p‐coumaric acid and quercetin) of adult worker honey bees (Liao et al., 2017a) and adult females of the yellow fever mosquito *Ae. aegypti* by quercetin (Nunes et al., 2016).

Our findings highlight a particularly pronounced effect of certain phytochemicals on the longevity of mosquitoes, which has important ramifications for understanding how mosquito fitness and vectorial capacity can be influenced by the presence of nectar sources in mosquito habitats. Additionally, results from our whole‐transcriptome analysis after consumption of nectar phytochemicals suggest further implications for insecticide resistance and mosquito–pathogen interactions.

## 2 MATERIALS AND METHODS

### 2.1 Mosquitoes for bioassays

All of the bioassays were conducted at the Medical Entomology Laboratory, Illinois Natural History Survey (INHS), University of Illinois at Urbana‐Champaign, using eggs of *Ae*. *albopictus* from a colony that was originally collected from Jacksonville, Florida. The adult mosquitoes were generated from a colony reared at 28°C, 80% relative humidity under a 16:8 hr photoperiod (light:dark cycle). Larvae were reared on lactalbumin:yeast (1:1) diet (Sigma‐Aldrich, St Louis, USA) in batches of approximately 100 larvae in 22.8 × 30.5 × 7.5 cm enamel pans.

### 2.2 Phytochemicals

Caffeine (CAS#58‐08‐2), *p*‐coumaric acid (CAS#501‐98‐4), and quercetin (CAS#117‐39‐5) were purchased from Sigma‐Aldrich Co. LLC., St. Louis, MO, USA, and used to prepare experimental solutions at concentrations within the natural range documented in nectar, honey, and pollen (Martos et al., 2000; Martos, Ferreres, Yao, et al., 2000; Serra Bonvehi et al., 2001; Wright et al., 2013; Kaškonienė et al., 2015; Mao et al., 2015; Cheung et al., 2019) of diverse plant species. Both quercetin and *p*‐coumaric acid were dissolved in dimethyl sulfoxide (DMSO; D128, Fisher Scientific International, Inc., Pittsburgh, PA, USA), whereas caffeine was dissolved in deionized (DI) water to make stock solutions. The dietary phytochemicals were prepared fresh from the stock solutions immediately before use. Anthrone reagent (CAS# 90‐44‐8) used in the cold‐anthrone test applied for the sugar‐feeding assays was purchased from Sigma‐Aldrich Co. LLC., St. Louis, MO, USA.

### 2.3 Longevity assays

Newly emerged adult female mosquitoes (1–3 days) were placed in paperboard cages (11 cm height × 9.5 cm diameter) in batches of 25 and fed ad libitum on diets of 10% sucrose containing either caffeine at 50, 100, or 200 ppm; *p*‐coumaric acid at 50, 100, or 200 ppm; or quercetin at 100, 200, or 400 ppm. The control group received 10% sucrose dissolved in deionized water and a solvent control with DMSO. We tested a wide range of concentrations of phytochemicals to determine whether any effects are concentration‐dependent. Each of the 11 treatment combinations was replicated four times, with 25 mosquitoes per replicate. Dead individuals were counted and removed from the cages daily.

### 2.4 Sugar‐feeding behavior

To determine the amount of sucrose consumed by female *Ae*. *albopictus* provided with 10% sucrose solution containing caffeine, *p*‐coumaric acid, or quercetin, visual quantification of ingested sucrose was carried out using the cold‐anthrone test (Haramis & Foster, 1983). Newly emerged female mosquitoes were placed in paperboard cages (11 cm height × 9.5 cm diameter) in batches of 25 and supplied with 10% sucrose solution for 24 hr, starved for 24 hr, and then provided with the diets described in the longevity assays for 3 hr using the highest concentration of each phytochemical. Each treatment was replicated four times, with 25 mosquitoes per replicate, yielding 500 experimental units. After 3 hr of feeding, individual mosquitoes were placed in a 1.5‐ml centrifuge tube and frozen at −80°C for quantification of the amount of sucrose consumed with anthrone. Anthrone solution (2 mg/ml) was prepared by dissolving 200 mg of anthrone reagent in 100 ml of 70% sulfuric acid.

The mosquitoes were thawed and moistened with 1:1 chloroform‐methanol solution (2 drops per mosquito) for 20 min to remove cuticular wax. The mosquitoes were then crushed gently with a glass rod and 1 ml of anthrone solution added to each test tube. The test tubes were vortexed and then incubated at 26°C in a water bath for 1 hr. The test tubes were agitated on a vortex mixer halfway through the hour and again at the end of the hour.

Standard sucrose solutions were prepared from 9 twofold serial dilutions of sucrose solutions corresponding to 1, 2, 4, 8, 16, 32, 64, 128, 256, and 512 µg/µl from an initial stock sucrose solution made by dissolving 51.2 g of sucrose in 100 ml of deionized water, and the resulting dilutions were mixed with anthrone solution. To quantify the amount of sucrose consumed by the mosquitoes, the color strength of each of the experimental tubes (mosquitoes fed with sucrose solutions containing the individual phytochemicals) was compared visually with the color strength of the standards prepared with known amounts of sucrose and designated according to the standard it most closely resembled.

### 2.5 Fecundity assays

Newly emerged female mosquitoes were maintained in the presence of males in a 1:1 sex ratio in paperboard cages (11 cm height × 9.5 cm diameter) and fed on diets of 10% sucrose containing: 200 ppm caffeine, 200 ppm *p*‐coumaric acid, 400 ppm quercetin, DI water (experimental control), or DMSO (solvent control) for 7 days. The females were then starved for 24 hr and thereafter exposed to a single blood meal of citrate‐buffered bovine blood (Hemostat Laboratories, Inc., Dixon, CA) using an artificial membrane feeder system (Hemotek Ltd., Blackburn, UK). Blood‐engorged female mosquitoes were immediately isolated in individual containers supplied with 10% sucrose solution containing a dietary phytochemical matching the prestarvation phytochemical to which they were initially exposed, and an oviposition cup. For each treatment, three trials comprising 35 gravid females per trial were conducted. Oviposition was monitored after 3 days, and eggs laid by individual mosquitoes were counted after 5 days. Mosquito body mass was used as a covariate because it is known to affect fecundity (Steinwascher, 1984) and was assessed by measuring the dry weight of the individual mosquitoes after egg‐laying. To measure the dry weight of the females, the mosquito specimens were dried at 40°C for 2 days and weighed on a Mettler M‐5 balance with a precision of ±0.005 mg.

### 2.6 Statistical analysis

For the longevity assays, the survival curves for the treatments were obtained with the Kaplan–Meier estimator in SPSS software (version 25.0; IBM Corp., Armonk, NY, USA). The difference in survival times of adult female *Ae*. *albopictus* feeding on the three dietary phytochemicals at different concentrations was compared by the log‐rank test with Bonferroni correction. For the sugar‐feeding assays, the differences in the amounts of sugars consumed across all treatments were analyzed by the nonparametric Kruskal–Wallis one‐way analysis of variance (ANOVA) in SPSS software (version 25.0; IBM Corp., Armonk, NY, USA). For fecundity assays, the data were log‐transformed and analyzed by two‐way analysis of covariance (ANCOVA) to determine the statistical differences in the number of eggs laid by mosquitoes across treatments.

### 2.7 Mosquito sugar‐feeding for RNA sequencing

Newly emerged female *Ae*. *albopictus* were placed in paperboard cages as described earlier in batches of 25 females/cage and fed ad libitum on treatments of 10% sucrose solution containing either 400 ppm quercetin or 200 ppm *p*‐coumaric acid. The control group received a 10% sucrose solution. The females were fed for 60 days; this time‐point was selected based on the results of the longevity assays. Each of the three treatments was replicated five times. After 60 days, individual mosquitoes from the three treatments were placed in 1.5‐ml centrifuge tubes, immediately flash‐frozen in liquid nitrogen, and stored in a −80°C freezer until RNA extraction.

### 2.8 RNA extraction, library construction, and RNA sequencing

One individual adult mosquito was randomly sampled from each of the five replicates per treatment and whole‐body RNA extracted using the NucleoSpin RNA® kit (Takara, Japan) according to the manufacturer's protocol. Recombinant DNase I (Takara, Japan) was used to remove potential genomic DNA. The integrity of RNA was analyzed on an Agilent 2100 Bioanalyzer (Agilent, Palo Alto, CA, USA) using an RNA 6000 nanochip. A total of 15 libraries were prepared individually using the five mosquitoes from each treatment. Libraries were prepared, quantified, and sequenced on two Sp lanes of an Illumina NovaSeq 6000 at the W.M. Keck Center for Comparative and Functional Genomics at the University of Illinois at Urbana‐Champaign to generate 150 bp paired‐end reads.

### 2.9 Quality control and mapping

The quality of the generated reads was assessed with FastQC (Andrews, 2010), and low‐quality and adaptor sequences were trimmed using trimmomatic (Bolger et al., 2014). The trimmed reads were mapped to the *Ae*. *albopictus* reference genome from the National Center for Biotechnology Information (NCBI), (https://www.ncbi.nlm.nih.gov/assembly/GCA_006516635.1) using STAR 2.6.0c (Dobin et al., 2013).

### 2.10 Analysis of differentially expressed (DE) genes

The resulting mapping files in bam format from the previous step were sorted and used to estimate transcript abundance with RSEM (Li & Dewey, 2011). These values were normalized via transcript per million (TPM). In addition, the transcript abundances were cross‐normalized using the Trimmed Means of M‐values (TMM) method (Li & Dewey, 2011). The TPM values were used to calculate differential gene expression between the three treatments with the R Bioconductor package EdgeR (Robinson et al., 2009). Transcripts with absolute fold change values ≥2.0 and false discovery rate (FDR) corrected *p*‐values <.05 were regarded as differentially expressed. Heat map figures were built with TPM‐normalized values using the “analyze_diff_expr.pl,” program from the Trinity package (Haas et al., 2013).

### 2.11 Gene ontology (GO) enrichment analysis and pathway enrichment analysis of DE genes

Predicted transcripts across the full genome were annotated with GO assignments utilizing the Trinotate annotation pipeline (v.2.0.2) as outlined by Haas et al. (2013) and served as the basis for examining term enrichment with the GOseq Bioconductor Package (Young et al., 2010) on the set of differentially expressed genes. Pathway enrichment analysis of DEs was performed via the Kyoto Encyclopedia of Genes and Genomes (KEGG). For that purpose, the KEGG automatic annotation server (Kanehisa et al., 2012) was used to run reciprocal best‐BLAST‐hit searches between the *Ae*. *albopictus* predicted transcripts and the transcript sets of 27 other organisms in the KEGG database, including arthropods and other model organisms for a wider representation of organisms. From the 43,354 mRNAs entered, 17,793 had orthologs in the KEGG database and were assigned a KEGG orthology (KO) number. The KO numbers for the differentially expressed genes for each of the comparisons were uploaded separately to query the KEGG reference pathways.

### 2.12 Quantitative RT‐PCR validation of RNA‐Seq data

To validate the results of differential gene expression detected with RNA‐Seq, ten candidate genes were selected from the most significantly differentially expressed genes to quantify their relative expression levels in the three treatments by quantitative real‐time polymerase chain reaction (qRT‐PCR). RNA was extracted from three pooled whole mosquitoes per replicate for all the three treatments as described for RNA‐Seq. Five biological replicates per treatment and three technical replicates per sample were applied. The cDNA was prepared from 0.62 μg of total RNA from each sample (New England BioLabs Inc., Massachusetts) following the manufacturer's protocol. The qRT‐PCRs for the selected genes were performed using the Luna^®^ Universal SYBR® Green qPCR Master Mix (New England BioLabs Inc., Massachusetts) in a 7300 FAST Real‐Time PCR System (ABI, Foster City, CA, USA) with the ribosomal protein 7 gene serving as an internal control (Kang et al., 2019). Gene‐specific qPCR primers were designed according to *Ae*. *albopictus* transcriptome sequences (Table S1). The relative expression level of each gene was calculated by the 2^−ΔΔCt^ method (Schmittgen & Livak, 2008). Relative expression values were assessed with *t* tests. The Pearson correlation coefficient was calculated between fold changes in transcript accumulation levels for *p*‐coumaric acid and quercetin treatments, as obtained by qRT‐PCR and RNA‐Seq, respectively.

## 3 RESULTS

### 3.1 Survival

**FIGURE 2:**
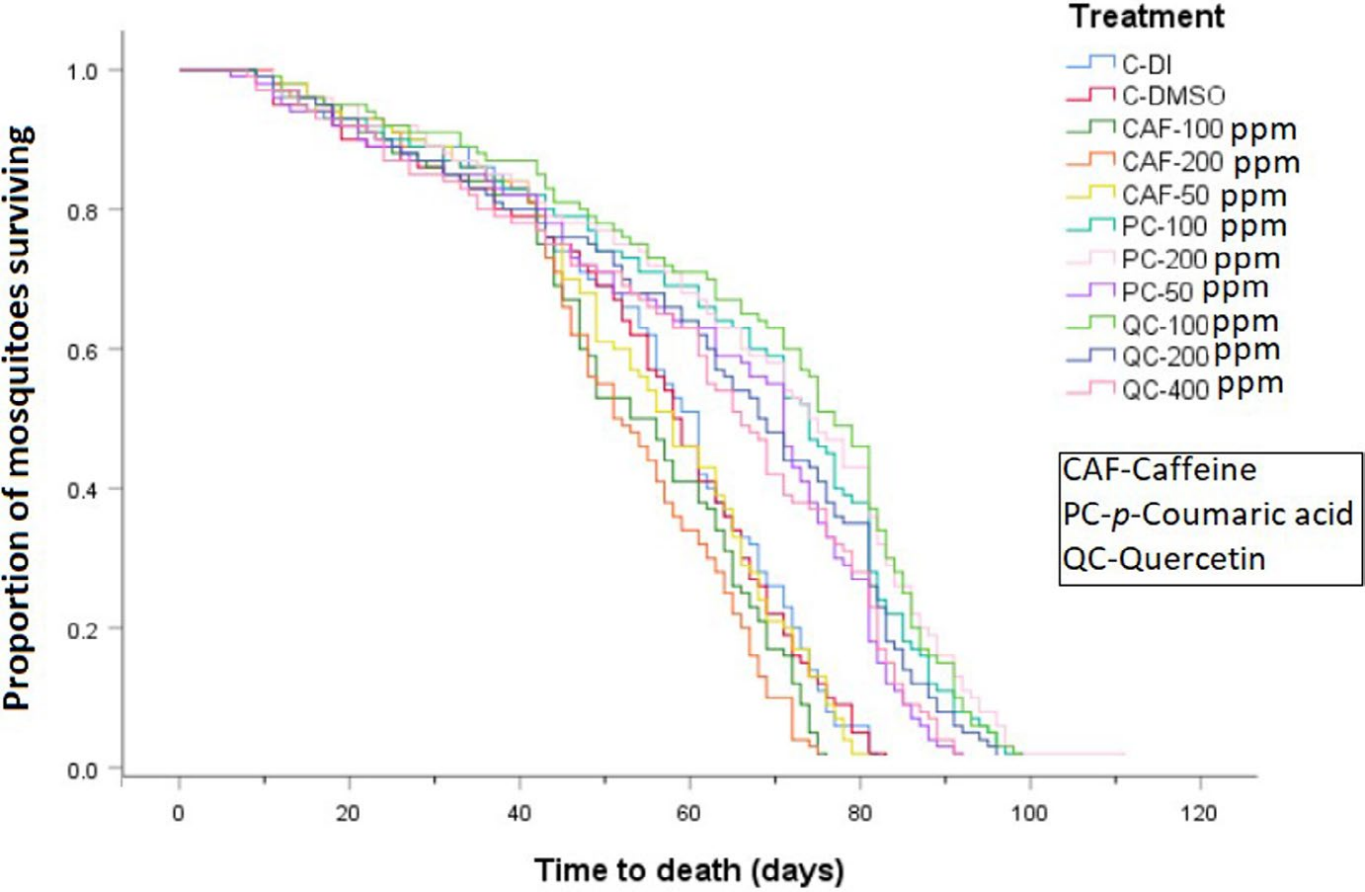
Kaplan–Meier survival plots of female *Ae*. *albopictus* consuming sucrose diets supplemented with phytochemicals (CAF) caffeine, PC (*p*‐coumaric acid) and QC (quercetin), treatment control (control‐DI), and solvent control (control‐DMSO). The concentrations are in parts per million (ppm). The survival curve comparisons with log‐rank (Mantel–Cox) test revealed significant differences in survival times (*p* < .001)

Survival times of female *Ae*. *albopictus* provided with dietary caffeine, *p‐*coumaric acid, or quercetin were influenced by the specific phytochemical consumed (χ^2^ = 243.06, *df* = 10, *p* < .001). Overall, mosquitoes receiving sucrose diets containing *p*‐coumaric acid and quercetin at all concentrations survived the longest compared with those consuming caffeine and control diets. The mosquitoes survived the longest on sucrose diets containing either 100 and 200 ppm *p*‐coumaric acid (median = 74 days), followed by 100 ppm quercetin, 50 ppm *p*‐coumaric acid, 200 and 400 ppm quercetin, controls, and caffeine in decreasing order (median = 72, 70, 69, 60, 58, 50, 58, and 51 days, respectively) (Figure 2 and Table 1). There were significant differences in survival times between mosquitoes consuming dietary *p*‐coumaric acid, quercetin, and the controls at all concentrations (Table S2). Generally, the survival times of mosquitoes consuming sucrose diets containing caffeine were comparable to survival times on control diets (Figure 2). There was no significant difference in survival time between the control and the 50‐ ppm caffeine diet, but higher concentrations (100 ppm and 200 ppm) of caffeine significantly reduced female *Ae*. *albopictus* survival time (Figure 2 and Table S2).

**TABLE 1:** Median and mean survival times (days) (± *SE*) for female *Ae*. *albopictus* consuming sucrose diets containing caffeine, *p‐*coumaric acid, or quercetin at different concentrations​

Phytochemical	Classification	Concentration (ppm)	*N*	Median	± *SE*	Mean	± *SE*
Caffeine	Alkaloid	50	100	58.00	2.49	54.70	1.79
		100	100	53.00	3.00	52.10	1.77
		200	100	51.00	2.73	51.13	1.61
*p‐*Coumaric acid	Phenolic acid	50	100	70.00	1.25	61.73	2.32
		100	100	74.00	2.50	65.78	2.39
		200	100	74.00	2.86	67.35	2.42
Quercetin	Flavonol	100	100	72.00	2.73	65.03	2.35
		200	100	69.00	2.69	66.05	2.37
		400	100	69.00	4.50	65.86	2.43
Control‐DI			100	60.00	1.54	55.95	1.81
Control‐DMSO			100	58.00	1.67	54.64	1.93

Note

The surviving mosquitoes from each treatment were censored on day 100.

Abbreviations: *N*, total number of female *Ae. albopictus* assayed; *SE*, standard error.

### 3.2 Sugar‐feeding behavior

**FIGURE 3:**
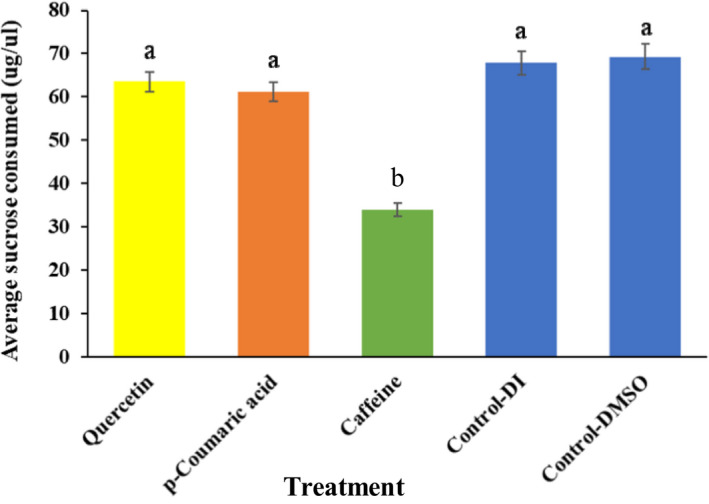
Mean (± *SE*) amount of sucrose consumed by female *Ae*. *albopictus* fed on dietary phytochemicals, caffeine (200 ppm), *p*‐coumaric acid (200 ppm), or quercetin (400 ppm), and control diets. Different lower‐case letters indicate statistical differences between treatments (Kruskal–Wallis one‐way ANOVA with Bonferroni correction)

There was a significant difference between treatments in the amount of sucrose consumed by female *Ae*. *albopictus* (χ^2^ = 155.942, *df* = 4, *p* < .001). Specifically, mosquitoes receiving a sucrose diet containing caffeine consumed significantly less sugar on average (33.84 μg/μl) compared with the other treatments (quercetin, 63.52 μg/μl; *p*‐coumaric acid, 61.12 μg/μl; or control, 67.84 μg/μl) (Figure 3). Dietary *p‐*coumaric and quercetin did not have a significant effect on the amount of sucrose ingested by female mosquitoes (Figure 3).

### 3.3 Fecundity

**FIGURE 4:**
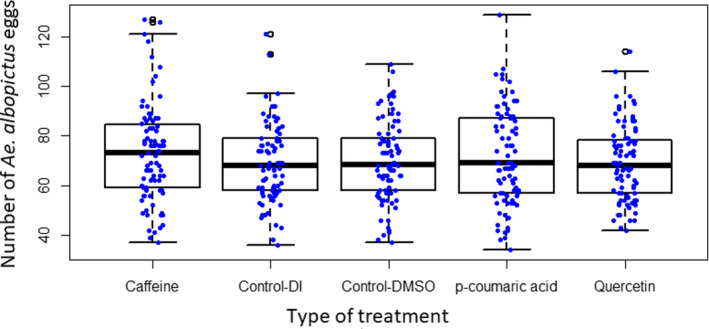
Distribution of the number of eggs laid by female *Ae*. *albopictus* feeding on sucrose diets containing caffeine (200 ppm), *p*‐coumaric acid (200 ppm), and quercetin (400 ppm), and control diets

After controlling for mosquito body weight, there was a significant difference in the number of eggs laid by female mosquitoes across the treatments (*F* = 4.906, *df* = 4, *p* = .001). Mosquito body weight had a significant effect on the number of eggs laid (*F* = 1,041.05, *df* = 1, *p* < .001). On average, female mosquitoes consuming sucrose diets containing caffeine laid a significantly greater number of eggs (73.03) compared with females consuming dietary *p*‐coumaric acid (71.38; *p* = .001), quercetin (69.02; *p* = .004), or control (69.53; *p* = .042) (Figure 4).

### 3.4 Effects of dietary *p*‐coumaric acid and quercetin on global gene expression

**FIGURE 5:**
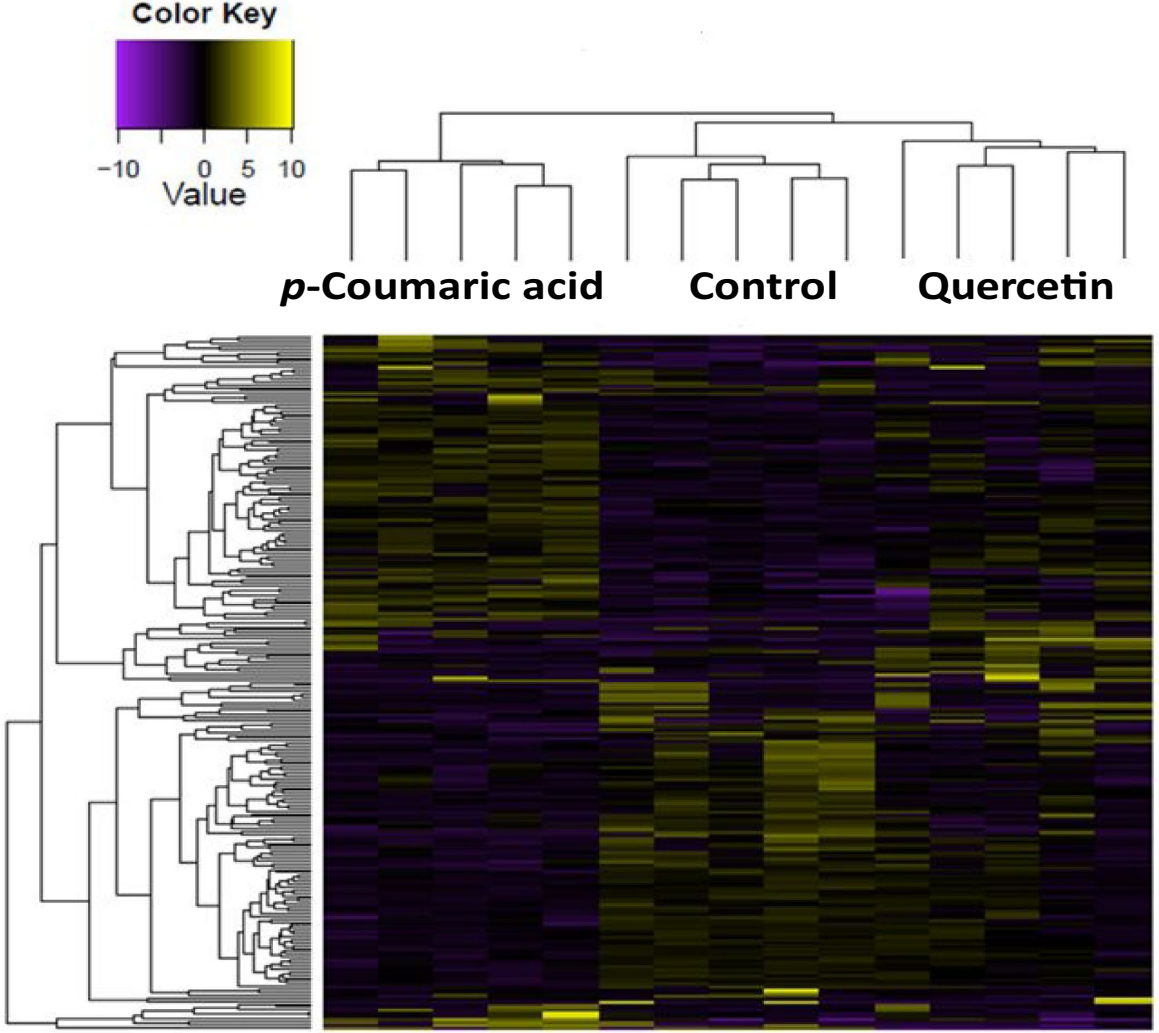
Heat map showing differentially expressed genes in female *Ae*. *albopictus* consuming sucrose diets supplemented with *p*‐coumaric acid or quercetin relative to control (sucrose‐only). The cutoff of log2 fold change 1.5 (>2.8‐fold change) and the FDR‐corrected *p*‐value <.05 was applied. Color key indicates the intensity associated with normalized values. Green shades indicate high expression and purple shades indicate low expression

**FIGURE 6:**
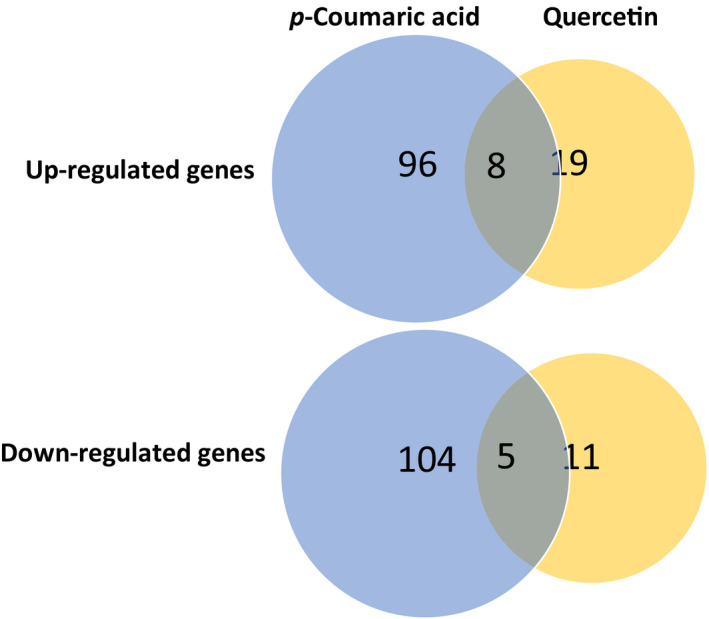
Differentially expressed genes in female *Ae*. *albopictus* consuming sucrose diets supplemented with *p*‐coumaric acid or quercetin. The cutoff for DE genes was log2 fold change 1.5 (>2.8‐fold change) and the FDR‐corrected *p*‐value <.05 was applied

The sequencing resulted in over 2 billion 150 bp paired‐end reads across 15 libraries (Table S3). On average per library, about 90% of reads were mapped to the reference *Ae*. *albopictus* genome, of which about 62% mapped uniquely, and the rest mapped to multiple locations. The biological replicates of control as well as *p*‐coumaric acid and quercetin treatments clustered closely, indicating that our sequencing data were qualified for identification of differentially expressed genes (Figures S1 and S2). Of the 29,586 predicted gene‐coding transcripts and isoform models (hereafter referred to as “genes”) in the reference genome, we recorded 237 that were significantly differentially expressed (DE) across pairwise treatment comparisons, with a cut‐off of log_2_ fold change of 1.5 (>2.8‐fold change) and FDR‐corrected *p*‐value <.05 (Figure 5 and Table S4). Compared with quercetin diet, consumption of dietary *p*‐coumaric acid by female *Ae*. *albopictus* changed the expression of more genes (Figure 5 and Table S4). In comparison with the control diet, a total of 96 genes were upregulated in female mosquitoes consuming dietary *p*‐coumaric acid and 19 genes in those consuming dietary quercetin. Additionally, 104 genes were downregulated with sucrose diets containing *p*‐coumaric acid and 11 genes with sucrose diets supplemented with quercetin (Figure 6).

Among genes significantly upregulated in the *p*‐coumaric acid treatment were several encoding enzymes associated with detoxification and antioxidant reactions. The transcript most highly upregulated in this treatment (>8 log2 fold change) represents a gene predictively annotated as “trans‐1,2‐dihydrobenzene‐1,2‐diol dehydrogenase‐like” (XM_019690081.2), an oxidoreductase enzyme involved in the metabolism of xenobiotics by cytochrome P450s in mammalian models (Maser, 1995). Other upregulated genes involved in antioxidant reactions were superoxide dismutase [Mn], mitochondrial‐like (XM_029861145.1), which was >6 log_2_ fold higher in the *p*‐coumaric acid compared with control, and microsomal glutathione S‐transferase 1‐like (XM_019694071.2). In addition, two genes coding for cytochrome P450s were also upregulated with *p*‐coumaric acid: CYP6Z23 (XM_020077410.2), and CYP12F19 (XM_019674571.2) (Table 2 and Table S4).

**TABLE 2:** Top 10 up‐ and down‐regulated genes in female *Ae*. *albopictus* consuming sucrose diets containing *p*‐coumaric acid. A cutoff of fold change ratio of ≥2 and *p*‐value <.05 was applied

Gene ID	Annotation	Log FC	FDR *p*‐value
**Up ‐ regulated genes**			
XM_029852718.1	Trans‐12‐dihydrobenzene‐12‐diol dehydrogenase‐like	−8.11	.0003
XM_029861005.1	Partner of xrn‐2 protein 1	−7.41	.0451
XM_019681290.2	Uncharacterized LOC109408065	−7.06	.0096
XM_029867165.1	Uncharacterized protein K02A2.6‐like	−7.02	<.001
XM_029861145.1	Superoxide dismutase [Mn], mitochondrial‐like	−6.97	.0161
XM_029879373.1	Uncharacterized LOC115270160	−6.91	.0106
XM_019697643.2	60S ribosomal protein L7a	−6.43	.0156
XM_019678289.2	Uncharacterized LOC109405252	−6.34	.0185
XM_029861914.1	Uncharacterized LOC115260698	−6.10	.0227
XM_019701391.2	Leucine‐rich repeat‐containing protein 1‐like	−5.38	.0385
**Down** **​‐** **regulated genes**			
XM_029854043.1	60S ribosomal protein L11	8.22	.0006
XM_019702731.2	60S ribosomal protein L3 transcript variant X1	8.00	.0054
XM_029868486.1	Histone H2A	7.62	<.001
XM_029877980.1	5′‐AMP‐protein kinase subunit gamma‐1‐like	7.39	<.001
XM_019704544.2	rutC family protein UK114‐like‐Molecular chaperone	6.91	.0176
XM_029868466.1	Histone H4	6.69	.0034
XM_029852177.1	Uncharacterized LOC109426110	6.65	.0433
XM_029877141.1	Carnosine N‐methyltransferase‐like	6.41	.0032
XM_029869063.1	Inhibitor of growth protein 4‐like transcript variant X1	6.41	.0275
XM_029872363.1	Subcomponent‐binding protein mitochondrial‐like	6.32	.0290

Genes downregulated with *p*‐coumaric acid consumption included multiple genes encoding histone proteins (Table 3). Additionally, several genes putatively encoding trafficking proteins were downregulated with *p*‐coumaric acid, including mitochondrial import inner membrane translocase subunit Tim29‐like (XM_029872092.1), protein transport protein Sec61 subunit beta (XM_019675629.2), trafficking protein particle complex subunit 4‐like (XM_029852578.1), and MFS‐type transporter SLC18B1‐like transcript variant X1 (XM_029872524.1) (Table 2 and Table S4).

**TABLE 3:** Downregulated genes encoding for histone proteins in female mosquitoes consuming dietary *p*‐coumaric acid and quercetin treatments

Gene ID	Annotation	Log FC	FDR *p*‐value
***p*** **‐Coumaric acid**			
XM_029868486.1	Histone H2A	7.62	<0.001
XM_029868466.1	Histone H4	6.69	0.0034
XM_029868509.1	Histone H4	4.55	0.0391
XM_019694407.3	Histone H4	4.82	0.0195
XM_019707432.2	Histone H2A	3.37	0.0049
XM_019694446.2	Histone H2A	3.15	0.0156
XM_029868498.1	Histone H2A‐like	3.08	0.0133
XM_019694445.2	Histone H2A	2.83	0.0265
XM_029868499.1	Histone H2B‐like	2.77	0.0185
XM_019707414.2	Histone H2B‐like	2.70	0.0234
XM_019707422.2	Histone H2B‐like	2.46	0.0275
XM_019707438.2	Histone H4	2.41	0.0185
XM_019707436.2	Histone H2A	2.40	0.0119
XM_019677400.2	Histone H2A	2.19	0.0442
XM_019670992.2	Histone H2B	1.94	0.0295
**Quercetin**			
XM_019694446.2	Histone H2A	3.38	0.0346
XM_019707438.2	Histone H4	2.59	0.0344
XM_019707436.2	Histone H2A	2.67	0.0047

Fewer DE genes were detected in females consuming dietary quercetin (Figures 5 and 6). Among the upregulated genes included trans‐1,2‐dihydrobenzene‐1,2‐diol dehydrogenase‐like (XM_019690081.2), acetylcholinesterase‐like (XM_019709882.2), and cytochrome c oxidase subunit 6B1‐like (XM_029878939.1) genes, whereas those significantly downregulated compared with the control included CYP6CB3 (XM_019674571.2) and genes coding for histones, as was the case for the *p*‐coumaric acid treatment (Table 4).

**TABLE 4:** Top 10 up‐ and downregulated genes in female *Ae*. *albopictus* receiving sucrose diets containing quercetin

Gene ID	Annotation	Log FC	FDR *p*‐value
**Up** **‐** **regulated genes**			
XM_019705586.2	40S ribosomal protein S21 transcript variant X1	−9.43	.0344
XM_029852718.1	Trans‐12‐dihydrobenzene‐12‐diol dehydrogenase‐like	−8.14	.0002
XM_029853907.1	Mite group 2 allergen Lep d 2‐like	−7.66	.0002
XM_019678289.2	Uncharacterized LOC109405252	−7.53	.0344
XM_019709882.2	Acetylcholinesterase‐like	−7.16	.0030
XM_019697643.2	60S ribosomal protein L7a	−6.84	.0378
XM_029856691.1	C‐type lectin 37Db‐like	−6.54	.0141
XM_029852798.1	U4/U6.U5 tri‐snRNP‐associated protein 2‐like	−5.86	.0347
XM_029853655.1	Uncharacterized LOC115255526	−4.59	.0007
XM_029878939.1	Cytochrome c oxidase subunit 6B1‐like	−4.43	.0002
**Down** **‐** **regulated genes**			
XM_019702731.2	60S ribosomal protein L3 transcript variant X1	8.32	.0217
XM_019691910.2	Mitochondrial import receptor TOM7 homolog	7.84	<.001
XM_019704187.2	COP9 signalosome complex subunit 5	5.95	.0330
XM_029871811.1	Metaxin‐2‐like transcript variant X1	4.53	.0007
XM_029863577.1	Dynein light chain roadblock‐type 2‐like	3.45	.0346
XM_019694446.2	Histone H2A	3.38	.0346
XM_019707436.2	Histone H2A	2.67	.0047
XM_019674571.2	CYP6CB3	2.60	.0347
XM_019707438.2	Histone H4	2.59	.0344
XM_019687856.2	Lamin Dm0‐like	2.41	.0107

Note

The cutoff of fold change ratio of ≥2 and *p*‐value <0.05 was applied.

Of special interest are genes that were differentially expressed in both *p*‐coumaric acid and quercetin treatments, given that the phenotypic effect of enhanced longevity was observed in both groups. There were eight genes upregulated in both treatments, including trans‐1,2‐dihydrobenzene‐1,2‐diol dehydrogenase‐like (XM_019690081.2), cytochrome c oxidase subunit 6B1‐like (XM_029878939.1), and microsomal glutathione S‐transferase 1‐like (XM_019694071.2) (Figure 6, Tables 2 and 4). Genes differentially expressed in both treatments, such as histones and those involved in xenobiotic metabolism, indicate their potential role in enhancing longevity of female *Ae*. *albopictus*.

Significant GO term enrichment was found in genes upregulated with *p*‐coumaric acid (FDR *p*‐value <.05) (Table 5). GO cellular component categories that showed overrepresentation were related mostly to DNA packaging and nucleosome organization and assembly, indicating that consuming *p*‐coumaric acid affected the structure of nucleosomes. In the GO biological process categories, enriched terms were again related to chromatin organization and nucleosome organization and assembly. Within the GO molecular functions, enriched terms were related to nucleic acid, heterocyclic compound binding, organic cyclic compound binding, and neurotrophin p75 receptor binding (Table 5).

**TABLE 5:** Go terms derived from upregulated genes overrepresented in female *Ae*. *albopictus* consuming dietary *p*‐coumaric acid (FDR *p*‐value <.05)

Term	Ontology	# of DE in the category	Over_represented_FDR
Nucleosome	Cellular component	17	0.000000
DNA packaging complex	Cellular component	17	0.000000
Protein–DNA complex	Cellular component	17	0.000000
Chromosomal part	Cellular component	24	0.000000
Nuclear nucleosome	Cellular component	5	0.007426
Lipid droplet	Cellular component	8	0.008056
Macromolecular complex	Cellular component	40	0.022605
Nucleosome assembly	Biological process	9	0.000337
Nucleosome organization	Biological process	9	0.002111
Protein–DNA complex assembly	Biological process	9	0.003030
Chromatin organization	Biological process	13	0.003030
Cellular macromolecular complex assembly	Biological process	15	0.006433
Protein–DNA complex subunit organization	Biological process	9	0.008056
Heterocyclic compound binding	Molecular Function	45	0.008056
Organic cyclic compound binding	Molecular Function	45	0.009642
Nucleic acid binding	Molecular Function	36	0.005404
Neurotrophin p75 receptor binding	Molecular Function	3	0.039215

The pathway analysis showed that *p*‐coumaric acid upregulated genes affecting 73 KEGG reference pathways in 31 subgroups, of which the carbohydrate metabolism, xenobiotic biodegradation and metabolism, signal transduction, and longevity regulation pathways had the highest gene counts. The five upregulated genes with homology to genes in the longevity‐regulating pathway of model organisms were all within the “dietary restriction” induced pathway for enhanced longevity (Figure S3). A disproportionate number of human reference disease pathways were affected (22 pathways in eight subgroups), but most of them had only one DE ortholog involved. The *p*‐coumaric acid downregulated set showed effects in 96 reference pathways, the main groups being amino acid metabolism, cell growth and death, immune system, and nervous system. Human disease subgroups were again present with 36 genes in nine subgroups (Table S5).

There were 19 reference pathways affected by genes upregulated with quercetin, with major representation in the xenobiotic biodegradation by the cytochrome P450 group. Only nine reference pathways were affected by genes downregulated with quercetin, most of which were within the human diseases category. Generally, the DE genes in both treatments affected key pathways such as longevity regulation (Figure S3), xenobiotic metabolism, cell death and growth, senescence, and human diseases (Table S5).

**FIGURE 7:**
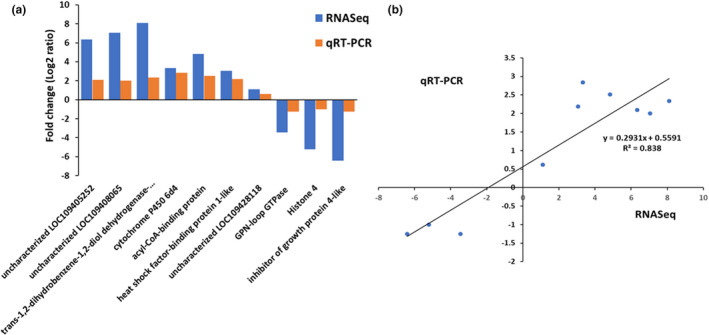
(a) Comparison of RNA‐Seq and qRT‐PCR results. RNA‐Seq and qRT‐PCR were based on fold changes in transcript levels. The *x*‐axis shows the 10 DE genes, while the *y*‐axis gives the degree of fold change for female *Ae*. *albopictus* consuming sucrose diet containing *p*‐coumaric acid. (b) Pearson correlation between fold changes in gene expression in female *Ae*. *albopictus*‐consuming dietary *p*‐coumaric acid treatment and on control diet, as determined by qRT‐PCR and RNA‐Seq (*p* < .05)

**FIGURE 8:**
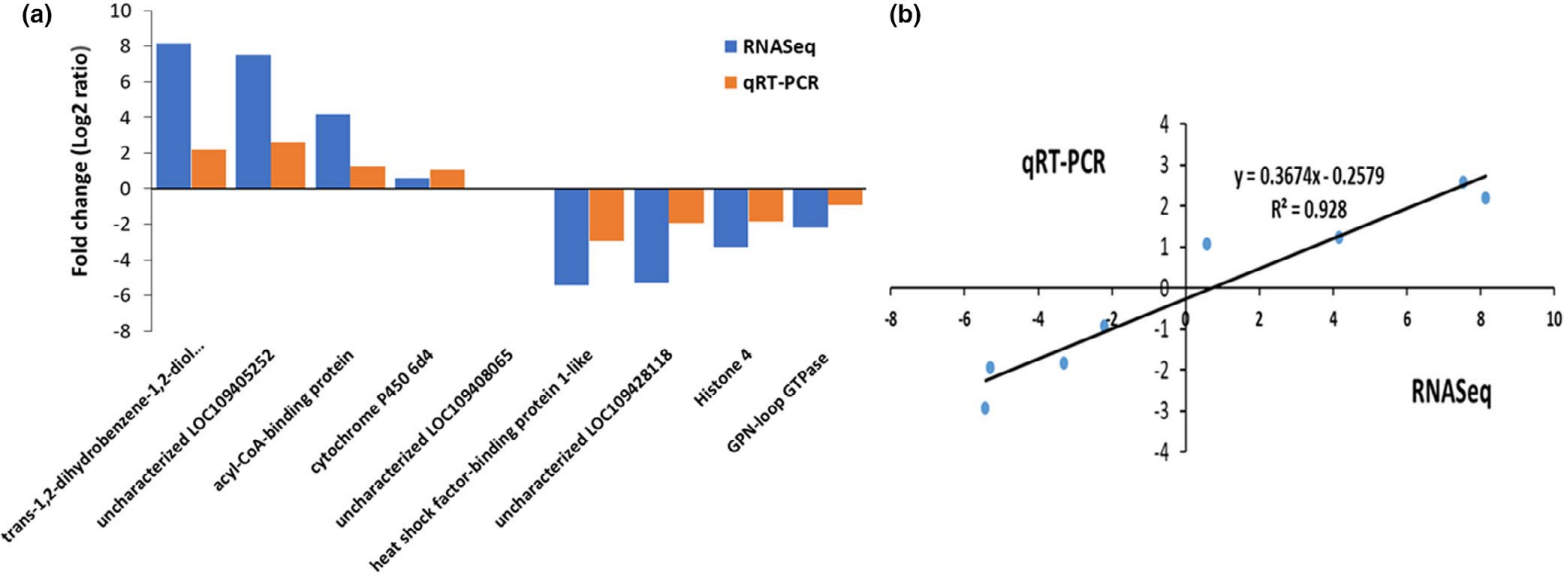
(a) Comparison of RNA‐Seq and qRT‐PCR results. RNA‐Seq and qRT‐PCR based fold changes in transcript levels. The *x*‐axis shows the 8 DE genes, while the *y*‐axis depicts the degree of fold change for female *Ae*. *albopictus* consuming sucrose diet containing quercetin. (b) Pearson correlation between fold changes in gene expression in female *Ae*. *albopictus* consuming dietary quercetin treatment and on control diet as determined by qRT‐PCR and RNA‐Seq (*p* < .05)

To validate the results obtained with the RNA‐Seq, the expression levels of several genes that showed significant differential expression in both *p*‐coumaric acid and quercetin treatments were quantified with qRT‐PCR. The qRT‐PCR results showed consistency in differential gene expression with RNA‐Seq data, and the correlation between the two methods was highly significant, with *R* ^2^ = 0.84 and *R* ^2^ = 0.93 for *p*‐coumaric acid and quercetin treatments, respectively (Figures 7 and 8).

## 4 DISCUSSION

In this study, we conducted laboratory assays and RNA sequencing to evaluate the ecological and physiological impacts of the nectar phytochemicals caffeine, *p*‐coumaric acid, and quercetin on adult female *Ae*. *albopictus*. Overall, our results revealed that the consumption of sucrose supplemented with certain phytochemicals enhanced longevity and fecundity, deterred sugar‐feeding, and changed the expression of genes involved in longevity regulation and xenobiotic metabolism, among others in female adult *Ae*. *albopictus*.

Dietary *p*‐coumaric acid and quercetin enhanced the longevity of the female mosquitoes, consistent with previous findings involving *Ae. aegypti* (Nunes et al., 2016) (for quercetin), honey bees (Bernklau et al., 2019; Liao et al., 2017a), and multiple model organisms, including *Drosophila melanogaster* and *Caenorhabditis elegans* (Kampkötter et al., 2008; Sunthonkun et al., 2019). Dietary *p*‐coumaric acid extended the life span of adult worker honey bees by 14.1% (Liao et al., 2017a) whereas quercetin enhanced the longevity of adult female *Ae. aegypti* by 30% (Nunes et al., 2016) and that of *C. elegans* by 15% (Kampkötter et al., 2008). The presence of these two phytochemicals in the diet did not affect the amount of sugar intake, suggesting that the extension of life span was not related to differential intake of sugars. These phytochemicals slightly affected fecundity, suggesting that the positive effect on life span was not due to a trade‐off between these two life‐history traits.

With the pronounced effects of enhanced mosquito longevity by *p*‐coumaric acid and quercetin, we conducted a whole‐transcriptome analysis to investigate the molecular processes that underlie the effects of the nectar phytochemicals on mosquito physiology and on vectorial attributes. At the transcriptome level, our results show that several differentially expressed genes in mosquitoes consuming dietary *p*‐coumaric acid and quercetin are related to pathways involving regulation of xenobiotic metabolism (Johnson et al., 2012; Mao et al., 2013), stress resistance (Zhang & Tsao, 2016), and longevity (Pallauf et al., 2017; Sunthonkun et al., 2019), demonstrating a possible contribution of the two phytochemicals in extending the life span of adult mosquitoes. Quercetin and *p*‐coumaric acid are antioxidants, and their role in longevity enhancement has been linked to their capacity to upregulate antioxidant enzymes that reduce the levels of reactive oxygen species (ROS) associated with aging in cells (Alugoju et al., 2018; Belinha et al., 2007; Kampkötter et al., 2008; Sunthonkun et al., 2019; Yue et al., 2019). We found that genes coding for two antioxidant enzymes, superoxide dismutase [Mn] mitochondria‐like (XM_029861145.1) and glutathione‐S‐transferases‐1 (GST1) (XM_019694071.2), were overexpressed in female mosquitoes consuming dietary quercetin and *p*‐coumaric acid. Manganese superoxide dismutase enzymes found in the mitochondria are known to protect cells against oxidative damage from toxic ROS associated with aging (Li & Zhou, 2011; Noblanc et al., 2020; Zhang et al., 2017).

The role of antioxidants in lifespan extension through enhanced resistance to oxidative stress has been demonstrated previously using model organisms such as yeast (*Saccharomyces cerevisiae*), *D. melanogaster,* and *C. elegans* as well as human epithelial cells (Ahn et al., 2014; Alugoju et al., 2018; Belinha et al., 2007; Kampkötter et al., 2008; Peng et al., 2018; Pietsch et al., 2009; Sunthonkun et al., 2019; Valenzano et al., 2006; Yue et al., 2019). In a study using yeast as a model, an ortholog of superoxide dismutase [Mn] mitochondria‐like, manganese dependent, superoxide dismutase 2 (SOD2), enhanced longevity, with quercetin increasing expression of the enzyme and effectively reducing the intracellular levels of ROS associated with aging (Sunthonkun et al., 2019). The phenolic acid *p‐*coumaric acid extended the life span of human epithelial cells and *C. elegans* by protecting the cells against oxidative stress associated with apoptosis (Peng et al., 2018; Yue et al., 2019). The role of heat shock proteins in counteracting proteotoxicity and oxidative stress that may underlie lifespan extension has been demonstrated in *D. melanogaster* and *C. elegans* (Lithgow & Walker, 2002; Morrow et al., 2004; Tower, 2011), and we found here that the heat shock factor‐binding protein 1‐like gene (XM‐029853002.1) was upregulated in female mosquitoes consuming dietary *p*‐coumaric acid and quercetin.

In both *p*‐coumaric acid and quercetin treatments, histone proteins were downregulated in the female mosquitoes. Changes in histones at the gene and protein level have been linked to cell death and senescence pathways in aging organisms. Histones are part of chromatin‐based processes in the nucleus, and they are major regulators of cellular and organismal aging. For instance, both loss of histones and change in expression levels are linked to aging in a mouse model and human fetal brain (Song & Johnson, 2018). The H2A subunit was overexpressed in senescent human fibroblasts, as well as in aging mice (Contrepois et al., 2017), in contrast with our finding, where this histone protein was downregulated with both *p*‐coumaric acid and quercetin, indicating its potential contribution in extending female *Ae*. *albopictus* life span.

The contribution of cytochrome P450s to the metabolism of natural products and synthetic xenobiotics in insects has been extensively evaluated (Feyereisen, 2006, 2011). In our study, three P450s were differentially expressed in female mosquitoes consuming dietary *p*‐coumaric acid or quercetin. Expression of CYP6Z23 (XM_01966080.2) was increased in females consuming dietary *p*‐coumaric acid. Several enzymes in the CYP6 subfamily in Diptera, including CYP6D4, CYP6Z1, CYP6N1, and CYP6M1, are associated with xenobiotic metabolism and insecticide resistance in *M*. *domestica* (house fly), *D. melanogaster,* and *Anopheles* and *Aedes* mosquitoes (Højland et al., 2014; Poupardin et al., 2010). Another P450 in the CYP6 family, CYP6CB3 (XM_019674571.2), was downregulated in both treatments. There is no gene annotation yet for this enzyme in *Ae*. *albopictus*. The expression of CYP12F19 (XM‐020077410.2) gene was increased with *p*‐coumaric acid but not with quercetin. Enzymes in the CYP12 subfamily in *Ae. aegypti,* including CYP12F7, are associated with pyrethroid resistance (Bariami et al., 2012; Faucon et al., 2015). The role of quercetin and *p*‐coumaric acid present in honey and pollen of angiosperms in enhancing pesticide tolerance and upregulation of P450 genes that metabolize pyrethroids and natural toxins has been documented previously in honey bees (Johnson et al., 2012; Liao et al., 2017a; Mao et al., 2009, 2015).

The XM_029852718.1 transcript encoding dihydrodiol dehydrogenase, an oxidoreductase enzyme involved in metabolism of xenobiotics by P450s (Schomburg et al., 1993), and the gene coding for cytochrome c oxidase subunit 6B1‐like (XM_029878939.1), an enzyme involved in cellular respiration and also contributes to antiaging effects in in vitro studies (Kim et al., 2015), were upregulated in both treatments. The acetylcholinesterase‐like gene (XM_019709882.2) was also upregulated in mosquitoes consuming dietary quercetin. This specialized carboxylic hydrolase is found at neuromuscular junctions where it terminates synaptic transmission, preventing continuous nerve firings at nerve endings (Lionetto et al., 2013) and is inhibited by organophosphates and carbamate pesticides (Hobbiger, 1961); its overexpression in female mosquitoes consuming dietary quercetin may have implications for insecticide resistance for vector species.

The gene (XM_029880481.1) encoding a UDP‐glucuronosyltransferase (UGT) was overexpressed in *Ae*. *albopictus* consuming dietary *p*‐coumaric acid. UGTs are multifunctional detoxification enzymes associated with degradation of xenobiotics (McGurk et al., 1998; Mehboob et al., 2017; Naydenova et al., 1999) and resistance to multiple insecticides in *Anopheles gambiae* (Vontas et al., 2005), *D*. *melanogaster* (Pedra et al., 2004), diamondback moth (*Plutella xylostella*) (Li et al., 2018) and African cotton leafworm (*Spodoptera littoralis*) Bozzolan et al., 2014). Additionally, the gene coding for a glutathione‐S‐transferase (GST1) (XM_019694071.2), belonging to a family of phase II detoxification enzymes, was upregulated in both treatments. GSTs are generally strongly implicated in resistance to multiple insecticides in mosquitoes (Hemingway et al., 2004). The activity of GST1 in the breakdown of xenobiotics and metabolites produced during cellular division and morphogenesis has been documented previously in *D. melanogaster* (Tu & Akgül, 2005). Apart from breakdown of toxic products, glutathione‐dependent enzymes are also involved in regulation of oxidative stress through ROS reduction, which is linked to enhanced longevity (Hayes & McLellan, 1999), indicating the potential contribution of GST1 in life span extension in female mosquitoes. We also found that the homologues of glutathione‐S‐transferases‐1, superoxide dismutase [Mn] mitochondria‐like, and the heat shock factor‐binding protein 1‐like genes (upregulated in the female mosquitoes consuming p‐coumaric acid and quercetin) are involved in the longevity‐regulating pathways of multiple model organisms, including mammals, flies, nematodes, and yeast.

The overexpression of genes involved in carbohydrate metabolism, such as “elongation of long‐chain fatty acids protein 4‐like” (XM_019672290.2) and “facilitated trehalose transporter Tret1‐like” (XM_029867481.1) in the female mosquitoes could play a role in the provision of sufficient energy to support lifespan extension. Genes that were downregulated in both *p*‐coumaric acid and quercetin treatments affected at least 20 KEGG reference pathways for human disease, including cancer and bacterial, viral, and neurodegenerative diseases. Although little is known about disease pathways in mosquitoes, our results indicate that *p*‐coumaric acid may affect longevity by directly affecting the expression of genes involved in disease and neural degeneration. *p‐*Coumaric acid and quercetin have anticancer and antimicrobial effects (Boz, 2015).

Consumption of caffeine at the highest concentration (100 ppm and 200 ppm) led to a significant reduction in adult female life span. Concentration‐dependent impacts of caffeine ingestion on longevity were reported in previous studies involving adult house flies (*Musca domestica*) (Srinivasan & Kesavan, 1979), *Drosophila prosaltans*, and *D. melanogaster* (Itoyama et al., 1998; Suh et al., 2017). By contrast, caffeine increased the life span of *C. elegans* at 15°C and 20°C in a temperature‐dependent lifespan extension study (Sutphin et al., 2012).

*Aedes albopictus* consumed less sucrose from solutions that contained caffeine, suggesting that it can act as a feeding deterrent. As a neuromodulator, caffeine exhibits both attractant and deterrent properties to foraging pollinators in a concentration‐dependent manner. High concentrations of caffeine (150 ppm and 200 ppm) comparable to levels used in our study repelled adult worker honey bees (Mustard et al., 2012; Singaravelan et al., 2005; Wright et al., 2013), possibly due to its bitter taste. The feeding deterrent property of caffeine in our study could explain the reduced life span in mosquitoes consuming a sucrose diet containing caffeine. A similar concentration of caffeine (200 ppm) supplied in sucrose diet, however, improved the memory of honey bee foragers, thereby enhancing flower visitation (Si et al., 2005; Wright et al., 2013). In two other separate studies, caffeinated nectar enhanced its quality, attracting more honey bees and leading to more efficient pollination (Couvillon et al., 2015; Thomson et al., 2015). Apart from concentration, the availability of alternative nectar sources also alters the deterrence of these compounds (Gegear et al., 2007; Stevenson et al., 2017).

Caffeine consumption slightly increased oviposition by female mosquitoes, inconsistent with previous findings involving *Ae. aegypti* assessing oviposition across multiple generations (Laranja et al., 2006). Moreover, in studies with insects other than mosquitoes, caffeine consumption reduced oviposition by the lepidopterans *Bombyx mori*, *Spodoptera litura*, *Danaus chrysippus,* and *Catopsilia crocale* (Mathavan et al., 1985), along with the dipteran *D*. *prosaltans* (Itoyama & Bicudo, 1992) and the hemipteran *Cimex lectularis* (Kamble & Narain, 2015).

In summary, this is the first study to evaluate the physiological impact of nectar phytochemicals on female adult mosquitoes at the molecular level. We demonstrated that *p*‐coumaric acid, and quercetin, present in nectars of many plant species, enhanced longevity, and altered the expression of genes involved in longevity regulation and xenobiotic metabolism in *Ae*. *albopictus*. We suggest that the lifespan extension capacity of *p*‐coumaric acid and quercetin is likely linked to the regulation of gene expression of life span‐related genes and xenobiotic metabolism. The phenolic acid *p*‐coumaric acid exerted a stronger effect and affected a wider range of genes than quercetin, possibly due to its ability to conjugate with small molecules that include sugars, which likely enhances its biological effects (Pei et al., 2016).

Our findings provide insights into the direct implications of nectar phytochemicals in the diet of adult female mosquitoes both at the organismal and molecular level by altering their sugar‐feeding behavior, fecundity, longevity, and gene expression. Our findings highlight that the role of phytochemicals needs to be considered when assessing how different nectar sources in the environment influence mosquito fitness, vectorial capacity, and, potentially, insecticide resistance. Future studies are needed to examine the effects of a wider range of nectar phytochemicals on the ecology of mosquito vectors, expanding the focus to encompass a greater diversity of vector species and the effect on vectorial capacity to establish whether enhancement of longevity by nectar feeding is a widespread feature of mosquito biology. Gene knockout studies are also needed to ascertain the phenotypic effects of differentially expressed genes affecting the longevity of mosquitoes.

## CONFLICT OF INTEREST

The authors have no conflict of interest.

## AUTHOR CONTRIBUTIONS

**Teresia M. Njoroge:** Conceptualization (equal); formal analysis (lead); methodology (lead); writing‐original draft (lead); writing‐review & editing (equal). **Bernarda Calla:** Conceptualization (equal); formal analysis (lead); methodology (equal); writing‐original draft (equal); writing‐review & editing (equal). **May R. Berenbaum:** Conceptualization (equal); funding acquisition (equal); methodology (equal); resources (equal); supervision (lead); writing‐original draft (equal); writing‐review & editing (equal). **Christopher M. Stone:** Conceptualization (equal); funding acquisition (equal); methodology (equal); resources (equal); supervision (lead); writing‐original draft (equal); writing‐review & editing (equal).

## Supporting information

Fig S1

Fig S2

Fig S3

Table S1‐S5

Supplementary Material

## Data Availability

All data from the study are either within the manuscript and in a supplemental file or a public repository at the NCBI Sequence Read Archive under the accession number PRJNA680162 (sequence reads).

## References

[ece37665-bib-0001] Adler, L. S. (2000). The ecological significance of toxic nectar. Oikos, 91, 409–420. 10.1034/j.1600-0706.2000.910301.x

[ece37665-bib-0002] Adler, L. S. , & Irwin, R. E. (2005). Ecological costs and benefits of defenses in nectar. Ecology, 86, 2968–2978. 10.1890/05-0118

[ece37665-bib-0003] Ahn, D. , Lee, E. B. , Kim, B. J. , Lee, S. Y. , Lee, T. G. , Ahn, M.‐S. , Lim, H. W. , Cha, D. S. , Jeon, H. , & Kim, D. K. (2014). Antioxidant and lifespan extending property of quercetin‐3‐O‐dirhamnoside from *Curcuma longa* L. in *Caenorhabditis elegans* . Journal of the Korean Society for Applied Biological Chemistry, 57, 709–714. 10.1007/s13765-014-4200-3

[ece37665-bib-0004] Alm, J. , Ohnmeiss, T. E. , Lanza, J. , & Vriesenga, L. (1990). Preference of cabbage white butterflies and honey bees for nectar that contains amino acids. Oecologia, 84(1), 53–57. 10.1007/BF00665594 28312774

[ece37665-bib-0005] Alugoju, P. , Janardhanshetty, S. S. , Subaramanian, S. , Periyasamy, L. , & Dyavaiah, M. (2018). Quercetin protects yeast *Saccharomyces cerevisiae* pep4 mutant from oxidative and apoptotic stress and extends chronological lifespan. Current Microbiology, 75, 519–530. 10.1007/s00284-017-1412-x 29224051

[ece37665-bib-0006] Andrews, S. (2010). FASTQC: A quality control tool for high throughput sequence data. http://www.bioinformatics.babraham.ac.uk/projects/fastqc/

[ece37665-bib-0007] Baracchi, D. , Brown, M. J. F. , & Chittka, L. (2015). Behavioural evidence for self‐medication in bumblebees? F1000Research, 4, 73. 10.12688/f1000research.6262.3 25949807 PMC4406194

[ece37665-bib-0008] Baracchi, D. , Marples, A. , Jenkins, A. J. , Leitch, A. R. , & Chittka, L. (2017). Nicotine in floral nectar pharmacologically influences bumblebee learning of floral features. Scientific Reports, 7, 1–8. 10.1038/s41598-017-01980-1 28512323 PMC5434031

[ece37665-bib-0009] Bariami, V. , Jones, C. M. , Poupardin, R. , Vontas, J. , & Ranson, H. (2012). Gene amplification, abc transporters and cytochrome p450s: Unraveling the molecular basis of pyrethroid resistance in the dengue vector, *Aedes aegypti* . PLoS Neglected Tropical Diseases, 6, e1692. 10.1371/journal.pntd.0001692 22720108 PMC3373657

[ece37665-bib-0010] Belinha, I. , Amorim, M. A. , Rodrigues, P. , De Freitas, V. , Moradas‐Ferreira, P. , Mateus, N. , & Costa, V. (2007). Quercetin increases oxidative stress resistance and longevity in *Saccharomyces cerevisiae* . Journal of Agricultural and Food Chemistry, 55, 2446–2451. 17323973 10.1021/jf063302e

[ece37665-bib-0011] Bennett, R. N. , & Wallsgrove, R. M. (1994). Secondary metabolites in plant defence mechanisms. New Phytologist, 127, 617–633. 10.1111/j.1469-8137.1994.tb02968.x 33874382

[ece37665-bib-0012] Bernklau, E. , Bjostad, L. , Hogeboom, A. , Carlisle, A. , & Arathi, H. S. (2019). Dietary phytochemicals, honey bee longevity and pathogen tolerance. Insects, 10(1), 14. 10.3390/insects10010014 PMC6359238 30626025

[ece37665-bib-0013] Bolger, A. M. , Lohse, M. , & Usadel, B. (2014). Trimmomatic: A flexible trimmer for Illumina sequence data. Bioinformatics, 30, 2114–2120. 10.1093/bioinformatics/btu170 24695404 PMC4103590

[ece37665-bib-0014] Boz, H. (2015). *p*‐Coumaric acid in cereals: Presence, antioxidant and antimicrobial effects. International Journal of Food Science & Technology, 50, 2323–2328.

[ece37665-bib-0015] Bozzolan, F. , Siaussat, D. , Maria, A. , Durand, N. , Pottier, M.‐A. , Chertemps, T. , & Maïbèche‐Coisne, M. (2014). Antennal uridine diphosphate (UDP)‐glycosyltransferases in a pest insect: Diversity and putative function in odorant and xenobiotics clearance. Insect Molecular Biology, 23, 539–549. 10.1111/imb.12100 24698447

[ece37665-bib-0016] Cheung, Y. , Meenu, M. , Yu, X. , & Xu, B. (2019). Phenolic acids and flavonoids profiles of commercial honey from different floral sources and geographic sources. International Journal of Food Properties, 22, 290–308. 10.1080/10942912.2019.1579835

[ece37665-bib-0017] Contrepois, K. , Coudereau, C. , Benayoun, B. A. , Schuler, N. , Roux, P. F. , Bischof, O. , & Mann, C. (2017). Histone variant H2A.J accumulates in senescent cells and promotes inflammatory gene expression. Nature Communications, 8, 14995. 10.1038/ncomms14995 PMC5436145 28489069

[ece37665-bib-0018] Couvillon, M. J. , Al Toufailia, H. , Butterfield, T. M. , Schrell, F. , Ratnieks, F. L. W. , & Schürch, R. (2015). Caffeinated forage tricks honeybees into increasing foraging and recruitment behaviors. Current Biology, 25, 2815–2818. 10.1016/j.cub.2015.08.052 26480843

[ece37665-bib-0019] Dobin, A. , Davis, C. A. , Schlesinger, F. , Drenkow, J. , Zaleski, C. , Jha, S. , Batut, P. , Chaisson, M. , & Gingeras, T. R. (2013). STAR: Ultrafast universal RNA‐seq aligner. Bioinformatics, 29, 15–21. 10.1093/bioinformatics/bts635 23104886 PMC3530905

[ece37665-bib-0020] Ebrahimi, B. , Jackson, B. T. , Guseman, J. L. , Przybylowicz, C. M. , Stone, C. M. , & Foster, W. A. (2018). Alteration of plant species assemblages can decrease the transmission potential of malaria mosquitoes. Journal of Applied Ecology, 55, 841–851. 10.1111/1365-2664.13001 PMC5849257 29551835

[ece37665-bib-0021] Faucon, F. , Dusfour, I. , Gaude, T. , Navratil, V. , Boyer, F. , Chandre, F. , & David, J. P. (2015). Identifying genomic changes associated with insecticide resistance in the dengue mosquito *Aedes aegypti* by deep targeted sequencing. Genome Research, 25, 1347–1359. 26206155 10.1101/gr.189225.115 PMC4561493

[ece37665-bib-0022] Feyereisen, R. (2006). Evolution of insect P450. Biochemical Society Transactions, 34, 1252–1255. 10.1042/BST0341252 17073796

[ece37665-bib-0023] Feyereisen, R. (2011). Arthropod CYPomes illustrate the tempo and mode in P450 evolution. Biochimica et Biophysica Acta ‐ Proteins and Proteomics, 1814, 19–28. 10.1016/j.bbapap.2010.06.012 20601227

[ece37665-bib-0024] Foster, W. A. (1995). Mosquito sugar feeding and reproductive energetics. Annual Review of Entomology, 40, 443–474. 10.1146/annurev.en.40.010195.002303 7810991

[ece37665-bib-0025] Gary, R. E. , & Foster, W. A. (2001). Effects of available sugar on the reproductive fitness and vectorial capacity of the malaria vector *Anopheles gambiae* (Diptera: Culicidae). Journal of Medical Entomology, 38, 22–28. 11268686 10.1603/0022-2585-38.1.22

[ece37665-bib-0026] Gary, R. E. , & Foster, W. A. (2004). *Anopheles gambiae* feeding and survival on honeydew and extra‐floral nectar of peridomestic plants. Medical and Veterinary Entomology, 18, 102–107. 10.1111/j.0269-283X.2004.00483.x 15189234

[ece37665-bib-0027] Gegear, R. J. , Manson, J. S. , & Thomson, J. D. (2007). Ecological context influences pollinator deterrence by alkaloids in floral nectar. Ecology Letters, 10, 375–382. 10.1111/j.1461-0248.2007.01027.x 17498136

[ece37665-bib-0028] Haas, B. J. , Papanicolaou, A. , Yassour, M. , Grabherr, M. , Blood, P. D. , Bowden, J. , Couger, M. B. , Eccles, D. , Li, B. O. , Lieber, M. , MacManes, M. D. , Ott, M. , Orvis, J. , Pochet, N. , Strozzi, F. , Weeks, N. , Westerman, R. , William, T. , Dewey, C. N. , … Regev, A. (2013). De novo transcript sequence reconstruction from RNA‐seq using the Trinity platform for reference generation and analysis. Nature Protocols, 8, 1494–1512. 10.1038/nprot.2013.084 23845962 PMC3875132

[ece37665-bib-0029] Hagler, J. R. , & Buchmann, S. L. (1993). Honey bee (Hymenoptera: Apidae) foraging responses to phenolic‐rich nectars. Journal of the Kansas Entomological Society, 223–230.

[ece37665-bib-0030] Haramis, L. D. , & Foster, W. A. (1983). Visual quantification of sugar in mosquitoes using anthrone reagent. Mosquito News, 43, 362–364.

[ece37665-bib-0031] Hayes, J. D. , & McLellan, L. I. (1999). Glutathione and glutathione‐dependent enzymes represent a co‐ordinately regulated defence against oxidative stress. Free Radical Research, 31, 273–300. 10.1080/10715769900300851 10517533

[ece37665-bib-0032] Hemingway, J. , Hawkes, N. J. , McCarroll, L. , & Ranson, H. (2004). The molecular basis of insecticide resistance in mosquitoes. Insect Biochemistry and Molecular Biology, 34, 653–665. 15242706 10.1016/j.ibmb.2004.03.018

[ece37665-bib-0033] Hien, D. F. D. S. , Dabiré, K. R. , Roche, B. , Diabaté, A. , Yerbanga, R. S. , Cohuet, A. , Yameogo, B. K. , Gouagna, L.‐C. , Hopkins, R. J. , Ouedraogo, G. A. , Simard, F. , Ouedraogo, J.‐B. , Ignell, R. , & Lefevre, T. (2016). Plant‐mediated effects on mosquito capacity to transmit human malaria. PLoS Path, 12, e1005773. 10.1371/journal.ppat.1005773 PMC4973987 27490374

[ece37665-bib-0034] Hobbiger, F. (1961). The inhibition of acetylcholinesterase by organophosphorus compounds and its reversal. Proceedings of the Royal Society of Medicine, 54, 403–405. 13714753 10.1177/003591576105400512 PMC1869569

[ece37665-bib-0035] Højland, D. H. , Jensen, K.‐M.‐V. , & Kristensen, M. (2014). Expression of xenobiotic metabolizing cytochrome P450 genes in a spinosad‐resistant *Musca domestica* L. strain. PLoS One, 9, e103689. 25165825 10.1371/journal.pone.0103689 PMC4148238

[ece37665-bib-0036] Impoinvil, D. E. , Kongere, J. O. , Foster, W. A. , Njiru, B. N. , Killeen, G. F. , Githure, J. I. , Beier, J. C. , Hassanali, A. , & Knols, B. G. J. (2004). Feeding and survival of the malaria vector *Anopheles gambiae* on plants growing in Kenya. Medical and Veterinary Entomology, 18, 108–115. 10.1111/j.0269-283X.2004.00484.x 15189235

[ece37665-bib-0037] Itoyama, M. M. , & Bicudo, H. E. M. D. (1992). Effects of caffeine on fecundity, egg‐laying capacity, development time and longevity in *Drosophila‐prosaltans* . Revista Brasileira de Genética, 15, 303–321.

[ece37665-bib-0038] Itoyama, M. M. , Bicudo, H. E. , & Manzato, A. J. (1998). The development of resistance to caffeine in *Drosophila prosaltans*: Productivity and longevity after ten generations of treatment. Cytobios, 96, 81–93. 10384710

[ece37665-bib-0039] Johnson, A. A. , & Riehle, M. A. (2015). Resveratrol fails to extend life span in the mosquito *Anopheles stephensi* . Rejuvenation Research, 18, 473–478. 25848933 10.1089/rej.2015.1670

[ece37665-bib-0040] Johnson, R. M. , Mao, W. , Pollock, H. S. , Niu, G. , Schuler, M. A. , & Berenbaum, M. R. (2012). Ecologically appropriate xenobiotics induce cytochrome P450s in *Apis mellifera* . PLoS One, 7, e31051. 10.1371/journal.pone.0031051 22319603 PMC3272026

[ece37665-bib-0041] Johnson, T. L. , Haque, U. , Monaghan, A. J. , Eisen, L. , Hahn, M. B. , Hayden, M. H. , Savage, H. M. , McAllister, J. , Mutebi, J.‐P. , & Eisen, R. J. (2017). Modeling the environmental suitability for *Aedes* (Stegomyia) *aegypti* and *Aedes* (Stegomyia) *albopictus* (Diptera: Culicidae) in the contiguous United States. Journal of Medical Entomology, 54, 1605–1614. 10.1093/jme/tjx163 29029153 PMC5868335

[ece37665-bib-0042] Kamble, S. T. , & Narain, R. B. (2015). Effects of ibuprofen and caffeine concentrations on the common bed bug (*Cimex lectulatius/em* L.) feeding and fecundity. Entomology, Ornithology & Herpetology: Current Research, 4, 2.

[ece37665-bib-0043] Kampkötter, A. , Timpel, C. , Zurawski, R. F. , Ruhl, S. , Chovolou, Y. , Proksch, P. , & Wätjen, W. (2008). Increase of stress resistance and lifespan of *Caenorhabditis elegan*s by quercetin. Comparative Biochemistry and Physiology ‐ B Biochemistry and Molecular Biology, 149, 314–323. 10.1016/j.cbpb.2007.10.004 18024103

[ece37665-bib-0044] Kanehisa, M. , Goto, S. , Sato, Y. , Furumichi, M. , & Tanabe, M. (2012). KEGG for integration and interpretation of large‐scale molecular data sets. Nucleic Acids Research, 40, 109–114. 10.1093/nar/gkr988 PMC3245020 22080510

[ece37665-bib-0045] Kang, S. , Shin, D. , Mathias, D. , Londono‐Renteria, B. , Noh, M. I. , Colpitts, T. , Dinglasan, R. , Han, Y. , & Hong, Y. (2019). Homologs of human dengue‐resistance genes, FKBP1B and ATCAY, confer antiviral resistance in *Aedes aegypti* mosquitoes. Insects, 10, 46. 10.3390/insects10020046 PMC6409984 30717390

[ece37665-bib-0046] Kaškonienė, V. , Ruočkuvienė, G. , Kaškonas, P. , Akuneca, I. , & Maruška, A. (2015). Chemometric analysis of bee pollen based on volatile and phenolic compound compositions and antioxidant properties. Food Analytical Methods, 8, 1150–1163. 10.1007/s12161-014-9996-2

[ece37665-bib-0047] Kim, S.‐E. , Mori, R. , Komatsu, T. , Chiba, T. , Hayashi, H. , Park, S. , & Shimokawa, I. (2015). Up‐regulation of cytochrome c oxidase subunit 6b1 (Cox6b1) and formation of mitochondrial supercomplexes: Implication of Cox6b1 in the effect of calorie restriction. AGE, 37, 45. 10.1007/s11357-015-9787-8 PMC4416092 25929654

[ece37665-bib-0048] Laranja, A. T. , Manzato, A. J. , & de Campos Bicudo, H. E. M. (2006). Caffeine effect on mortality and oviposition in successive generations of *Aedes aegypti* . Revista de Saude Publica, 40, 1112–1117. 10.1590/S0034-89102006000700022 17173171

[ece37665-bib-0049] Li, B. , & Dewey, C. N. (2011). RSEM: Accurate transcript quantification from RNA‐Seq data with or without a reference genome. BMC Bioinformatics, 12, 323. 10.1186/1471-2105-12-323 21816040 PMC3163565

[ece37665-bib-0050] Li, C. , & Zhou, H. M. (2011). The role of manganese superoxide dismutase in inflammation defense. Enzyme Research, 2011, 1–6. 10.4061/2011/387176 PMC3185262 21977313

[ece37665-bib-0051] Li, X. , Shi, H. , Gao, X. , & Liang, P. (2018). Characterization of UDP‐glucuronosyltransferase genes and their possible roles in multi‐insecticide resistance in *Plutella xylostella* (L.). Pest Management Science, 74, 695–704. 29027758 10.1002/ps.4765

[ece37665-bib-0052] Liao, L.‐H. , Wu, W.‐Y. , & Berenbaum, M. (2017a). Impacts of dietary phytochemicals in the presence and pbsence of pesticides on longevity of honey bees (*Apis mellifera*). Insects, 8, 22. 10.3390/insects8010022 PMC5371950 28216580

[ece37665-bib-0053] Liao, L. H. , Wu, W. Y. , & Berenbaum, M. R. (2017b). Behavioral responses of honey bees (*Apis mellifera*) to natural and synthetic xenobiotics in food. Scientific Reports, 7, 1–8. 10.1038/s41598-017-15066-5 29162843 PMC5698444

[ece37665-bib-0054] Lin Liu, F. , Jun Fu, W. , Rong Yang, D. A. , Peng, Y. Q. , Zhang, X. W. , & He, J. Z. (2004). Reinforcement of bee—plant interaction by phenolics in food. Journal of Apicultural Research, 43, 155–157. 10.1080/00218839.2004.11101128

[ece37665-bib-0055] Lionetto, M. G. , Caricato, R. , Calisi, A. , Giordano, M. E. , & Schettino, T. (2013). Acetylcholinesterase as a biomarker in environmental and occupational medicine: New insights and future perspectives. BioMed Research International, 2013, 321213. 10.1155/2013/321213 23936791 PMC3727120

[ece37665-bib-0056] Lithgow, G. J. , & Walker, G. A. (2002). Stress resistance as a determinate of *C. elegans* lifespan. Mechanisms of Ageing and Development, 123, 765–771. 10.1016/S0047-6374(01)00422-5 11869734

[ece37665-bib-0057] Liu, F. , Chen, J. , Chai, J. , Zhang, X. , Bai, X. , He, D. , & Roubik, D. W. (2007). Adaptive functions of defensive plant phenolics and a non‐linear bee response to nectar components. Functional Ecology, 21, 96–100. 10.1111/j.1365-2435.2006.01200.x

[ece37665-bib-0058] Manda, H. , Gouagna, L. C. , Foster, W. A. , Jackson, R. R. , Beier, J. C. , Githure, J. I. , & Hassanali, A. (2007). Effect of discriminative plant‐sugar feeding on the survival and fecundity of *Anopheles gambiae* . Malaria Journal, 6, 113. 10.1186/1475-2875-6-113 17711580 PMC2034389

[ece37665-bib-0059] Mao, W. , Rupasinghe, S. G. , Johnson, R. M. , Zangerl, A. R. , Schuler, M. A. , & Berenbaum, M. R. (2009). Quercetin‐metabolizing CYP6AS enzymes of the pollinator *Apis mellifera* (Hymenoptera: Apidae). Comparative Biochemistry and Physiology Part B: Biochemistry and Molecular Biology, 154, 427–434. 10.1016/j.cbpb.2009.08.008 19737624

[ece37665-bib-0060] Mao, W. , Schuler, M. A. , & Berenbaum, M. R. (2013). Honey constituents up‐regulate detoxification and immunity genes in the western honey bee *Apis mellifera* . Proceedings of the National Academy of Sciences of the United States of America, 110, 8842–8846. 10.1073/pnas.1303884110 23630255 PMC3670375

[ece37665-bib-0061] Mao, W. , Schuler, M. A. , & Berenbaum, M. R. (2015). A dietary phytochemical alters caste‐associated gene expression in honey bees. Science Advances, 1, e1500795. 10.1126/sciadv.1500795 26601244 PMC4643792

[ece37665-bib-0062] Martos, I. , Ferreres, F. , & Tomás‐Barberán, F. A. (2000). Identification of flavonoid markers for the botanical origin of *Eucalyptus* honey. Journal of Agricultural and Food Chemistry, 48, 1498–1502. 10820049 10.1021/jf991166q

[ece37665-bib-0063] Martos, I. , Ferreres, F. , Yao, L. , D'Arcy, B. , Caffin, N. , & Tomas‐Barberan, F. A. (2000). Flavonoids in monospecific Eucalyptus honeys from Australia. Journal of Agricultural and Food Chemistry, 48, 4744–4748. 11052728 10.1021/jf000277i

[ece37665-bib-0064] Maser, E. (1995). Xenobiotic carbonyl reduction and physiological steroid oxidoreduction. The pluripotency of several hydroxysteroid dehydrogenases. Biochemical Pharmacology, 49, 421–440. 10.1016/0006-2952(94)00330-O 7872949

[ece37665-bib-0065] Mathavan, S. , Premalatha, Y. , & Christopher, M. S. (1985). Effects of caffeine and theophylline on the fecundity of four lepidopteran species. Experimental Biology, 44, 133–138. 3850026

[ece37665-bib-0066] McGurk, K. A. , Brierley, C. H. , & Burchell, B. (1998). Drug glucuronidation by human renal UDP‐glucuronosyltransferases. Biochemical Pharmacology, 55, 1005–1012. 9605424 10.1016/s0006-2952(97)00534-0

[ece37665-bib-0067] Mehboob, H. , Tahir, I. M. , Iqbal, T. , Akhter, N. , Munir, N. , & Riaz, M. (2017). Genetic polymorphism of UDP‐glucuronosyltransferase. In N. Parine (Ed.),Genetic Polymorphisms, (pp. 159). Rijeka, Croatia: IntechOpen.

[ece37665-bib-0068] Mevi‐Schütz, J. , & Erhardt, A. (2005). Amino acids in nectar enhance butterfly fecundity: A long‐awaited link. American Naturalist, 165, 411–419. 10.1086/429150 15791533

[ece37665-bib-0069] Morrow, G. , Samson, M. , Michaud, S. , & Tanguay, R. M. (2004). Overexpression of the small mitochondrial Hsp22 extends Drosophila life span and increases resistance to oxidative stress. The FASEB Journal: Official Publication of the Federation of American Societies for Experimental Biology, 18, 598–599. 14734639 10.1096/fj.03-0860fje

[ece37665-bib-0070] Müller, G. C. , Xue, R. D. , & Schlein, Y. (2011). Differential attraction of *Aedes albopictus* in the field to flowers, fruits and honeydew. Acta Tropica, 118, 45–49. 10.1016/j.actatropica.2011.01.009 21310142

[ece37665-bib-0071] Mustard, J. A. , Dews, L. , Brugato, A. , Dey, K. , & Wright, G. A. (2012). Consumption of an acute dose of caffeine reduces acquisition but not memory in the honey bee. Behavioural Brain Research, 232, 217–224. 10.1016/j.bbr.2012.04.014 22521838

[ece37665-bib-0072] Naydenova, Z. , Krauss, G. J. , Golovinsky, E. , & Grancharov, K. (1999). Effect of s‐triazine and phenoxyalkanoic acid herbicides on UDP‐ glucuronosyltransferase in rat liver microsomes. Pesticide Science, 55, 825–830. 10.1002/ps.2780550809

[ece37665-bib-0073] Nepi, M. , Grasso, D. A. , & Mancuso, S. (2018). Nectar in plant–insect mutualistic relationships: From food reward to partner manipulation. Frontiers in Plant Science, 9, 1063. 10.3389/fpls.2018.01063 30073014 PMC6060274

[ece37665-bib-0074] Nicolson, S. W. , & Thornburg, R. W. (2007). Nectar chemistry. In S. W. Nicolson , M. Nepi , & E. Pacini (Eds.), Nectaries and nectar (pp. 215–264). Dordrecht, Netherlands: Springer. 10.1007/978-1-4020-5937-7_5

[ece37665-bib-0075] Nikbakhtzadeh, M. R. , Terbot, J. W. II , & Foster, W. A. (2016). Survival value and sugar access of four East African plant species attractive to a laboratory strain of sympatric *Anopheles gambiae* (Diptera: Culicidae). Journal of Medical Entomology, 53, 1105. 27247348 10.1093/jme/tjw067 PMC5013815

[ece37665-bib-0076] Nikbakhtzadeh, M. R. , Terbot, J. W. , Otienoburu, P. E. , & Foster, W. A. (2014). Olfactory basis of floral preference of the malaria vector *Anopheles gambiae* (Diptera: Culicidae) among common African plants. Journal of Vector Ecology, 39, 372–383. 25424267 10.1111/jvec.12113

[ece37665-bib-0077] Noblanc, A. , Klaassen, A. , & Robaire, B. (2020). The exacerbation of aging and oxidative stress in the epididymis of sod1 null mice. Antioxidants, 9, 151. 10.3390/antiox9020151 PMC7071042 32054065

[ece37665-bib-0078] Nunes, R. D. , Ventura‐Martins, G. , Moretti, D. M. , Medeiros‐Castro, P. , Rocha‐Santos, C. , Daumas‐Filho, C. R. D. O. , Bittencourt‐Cunha, P. R. B. , Martins‐Cardoso, K. , Cudischevitch, C. O. , Menna‐Barreto, R. F. S. , Oliveira, J. H. M. , Gusmão, D. S. , Alves Lemos, F. J. , Alviano, D. S. , Oliveira, P. L. , Lowenberger, C. , Majerowicz, D. , Oliveira, R. M. , Mesquita, R. D. , … Silva‐Neto, M. A. C. (2016). Polyphenol‐rich diets exacerbate AMPK‐mediated autophagy, decreasing proliferation of mosquito midgut microbiota, and extending vector lifespan. PLoS Neglected Tropical Diseases, 10, e0005034. 10.1371/journal.pntd.0005034 27732590 PMC5061323

[ece37665-bib-0079] Nyasembe, V. O. , Cheseto, X. , Kaplan, F. , Foster, W. A. , Teal, P. E. A. , Tumlinson, J. H. , Borgemeister, C. , & Torto, B. (2015). The invasive American weed *Parthenium hysterophorus* can negatively impact malaria control in Africa. PLoS One, 10, e0137836. 10.1371/journal.pone.0137836 26367123 PMC4569267

[ece37665-bib-0080] Nyasembe, V. O. , Teal, P. E. , Mukabana, W. R. , Tumlinson, J. H. , & Torto, B. (2012). Behavioural response of the malaria vector *Anopheles gambiae* to host plant volatiles and synthetic blends. Parasites and Vectors, 5, 1–11. 10.1186/1756-3305-5-234 23069316 PMC3523964

[ece37665-bib-0081] Ollerton, J. , Winfree, R. , & Tarrant, S. (2011). How many flowering plants are pollinated by animals? Oikos, 120, 321–326.

[ece37665-bib-0082] Pallauf, K. , Duckstein, N. , & Rimbach, G. (2017). A literature review of flavonoids and lifespan in model organisms. Proceedings of the Nutrition Society, 76, 145–162. 10.1017/S0029665116000720 27609098

[ece37665-bib-0083] Palmer‐Young, E. C. , Tozkar, C. Ö. , Schwarz, R. S. , Chen, Y. , Irwin, R. E. , Adler, L. S. , & Evans, J. D. (2017). Nectar and pollen phytochemicals stimulate honey bee (Hymenoptera: Apidae) immunity to viral infection. Journal of Economic Entomology, 110, 1959–1972. 10.1093/jee/tox193 28981688

[ece37665-bib-0084] Paupy, C. , Delatte, H. , Bagny, L. , Corbel, V. , & Fontenille, D. (2009). *Aedes albopictus*, an arbovirus vector: From the darkness to the light. Microbes and Infection, 11, 1177–1185. 10.1016/j.micinf.2009.05.005 19450706

[ece37665-bib-0085] Peach, D. A. H. , & Gries, G. (2016). Nectar thieves or invited pollinators? A case study of tansy flowers and common house mosquitoes. Arthropod‐Plant Interactions, 10, 497–506. 10.1007/s11829-016-9445-9

[ece37665-bib-0086] Peach, D. A. H. , & Gries, G. (2020). Mosquito phytophagy‐sources exploited, ecological function, and evolutionary transition to haematophagy. Entomologia Experimentalis et Applicata, 168, 120–136. 10.1111/eea.12852

[ece37665-bib-0087] Pedra, J. H. F. , McIntyre, L. M. , Scharf, M. E. , & Pittendrigh, B. R. (2004). Genome‐wide transcription profile of field‐ and laboratory‐selected dichlorodiphenyltrichloroethane (DDT)‐resistant *Drosophila* . Proceedings of the National Academy of Sciences of the United States of America, 101, 7034–7039. 10.1073/pnas.0400580101 15118106 PMC406461

[ece37665-bib-0088] Pei, K. , Ou, J. , Huang, J. , & Ou, S. (2016). *p*‐Coumaric acid and its conjugates: Dietary sources, pharmacokinetic properties and biological activities. Journal of the Science of Food and Agriculture, 96, 2952–2962. 26692250 10.1002/jsfa.7578

[ece37665-bib-0089] Peng, J. , Zheng, T.‐T. , Liang, Y. , Duan, L.‐F. , Zhang, Y.‐D. , Wang, L.‐J. , & Xiao, H.‐T. (2018). *p*‐Coumaric acid protects human lens epithelial cells against oxidative stress‐induced apoptosis by MAPK signaling. Oxidative Medicine and Cellular Longevity, 2018, 8549052. 29849919 10.1155/2018/8549052 PMC5914090

[ece37665-bib-0090] Pietsch, K. , Saul, N. , Menzel, R. , Stürzenbaum, S. R. , & Steinberg, C. E. W. (2009). Quercetin mediated lifespan extension in *Caenorhabditis elegans* is modulated by age‐1, daf‐2, sek‐1 and unc‐43. Biogerontology, 10, 565–578. 10.1007/s10522-008-9199-6 19043800

[ece37665-bib-0091] Poupardin, R. , Riaz, M. A. , Vontas, J. , David, J. P. , & Reynaud, S. (2010). Transcription profiling of eleven cytochrome P450s potentially involved in xenobiotic metabolism in the mosquito *Aedes aegypti* . Insect Molecular Biology, 19, 185–193. 20041961 10.1111/j.1365-2583.2009.00967.x

[ece37665-bib-0092] Richardson, L. L. , Adler, L. S. , Leonard, A. S. , Andicoechea, J. , Regan, K. H. , Anthony, W. E. , & Irwin, R. E. (2015). Secondary metabolites in floral nectar reduce parasite infections in bumblebees. Proceedings of the Royal Society B: Biological Sciences, 282, 20142471. 10.1098/rspb.2014.2471 PMC4345440 25694627

[ece37665-bib-0093] Rivera‐Pérez, C. , Clifton, M. E. , & Noriega, F. G. (2017). How micronutrients influence the physiology of mosquitoes. Current Opinion in Insect Science, 23, 112–117. 10.1016/j.cois.2017.07.002 29129275 PMC5695569

[ece37665-bib-0094] Robinson, M. D. , McCarthy, D. J. , & Smyth, G. K. (2009). edgeR: A Bioconductor package for differential expression analysis of digital gene expression data. Bioinformatics, 26, 139–140. 10.1093/bioinformatics/btp616 19910308 PMC2796818

[ece37665-bib-0095] Schmittgen, T. D. , & Livak, K. J. (2008). Analyzing real‐time PCR data by the comparative CT method. Nature Protocols, 3, 1101–1108. 10.1038/nprot.2008.73 18546601

[ece37665-bib-0096] Schomburg, D. , Salzmann, M. , & Stephan, D. (1993). trans‐1,2‐Dihydrobenzene‐1,2‐diol dehydrogenase. In D. Schomburg , M. Salzmann , & D. Stephan (Eds.), Enzyme handbook (pp. 443–448). Berlin, Heidelberg: Springer.

[ece37665-bib-0097] Serra Bonvehi, J. , Soliva Torrentó, M. , & Centelles Lorente, E. (2001). Evaluation of polyphenolic and flavonoid compounds in honeybee‐collected pollen produced in Spain. Journal of Agricultural and Food Chemistry, 49, 1848–1853. 10.1021/jf0012300 11308335

[ece37665-bib-0098] Si, A. , Zhang, S. W. , & Maleszka, R. (2005). Effects of caffeine on olfactory and visual learning in the honey bee (*Apis mellifera*). Pharmacology Biochemistry and Behavior, 82, 664–672. 10.1016/j.pbb.2005.11.009 16375953

[ece37665-bib-0099] Singaravelan, N. , Nee'man, G. , Inbar, M. , & Izhaki, I. (2005). Feeding responses of free‐flying honeybees to secondary compounds mimicking floral nectars. Journal of Chemical Ecology, 31, 2791–2804. 10.1007/s10886-005-8394-z 16365705

[ece37665-bib-0100] Song, S. , & Johnson, F. B. (2018). Epigenetic mechanisms impacting aging: A focus on histone levels and telomeres. Genes, 9, 201. 10.3390/genes9040201 PMC5924543 29642537

[ece37665-bib-0101] Srinivasan, A. , & Kesavan, P. C. (1979). Effect of caffeine on longevity and reproduction of the housefly. Toxicology Letters, 3, 101–105. 10.1016/0378-4274(79)90093-6

[ece37665-bib-0102] Steinwascher, K. (1984). Egg size variation in *Aedes aegypti*: Relationship to body s ize and other variables. American Midland Naturalist, 112, 76. 10.2307/2425459

[ece37665-bib-0103] Stevenson, P. C. , Nicolson, S. W. , & Wright, G. A. (2017). Plant secondary metabolites in nectar: Impacts on pollinators and ecological functions. Functional Ecology, 31, 65–75. 10.1111/1365-2435.12761

[ece37665-bib-0104] Stone, C. , & Foster, W. (2013). Plant‐sugar feeding and vectorial capacity. In T. Willem , & K. Sander (Eds.), Ecology of parasite‐vector interactions (pp. 35–79). Wageningen Academic Publishers.

[ece37665-bib-0105] Stone, C. M. , Jackson, B. T. , & Foster, W. A. (2012). Effects of plant‐community composition on the vectorial capacity and fitness of the malaria mosquito *Anopheles gambiae* . American Journal of Tropical Medicine and Hygiene, 87, 727–736. 10.4269/ajtmh.2012.12-0123 PMC3516327 22927493

[ece37665-bib-0106] Suh, H. J. , Shin, B. , Han, S. H. , Woo, M. J. , & Hong, K. B. (2017). Behavioral changes and survival in *Drosophila melanogaster*: Effects of ascorbic acid, taurine, and caffeine. Biological and Pharmaceutical Bulletin, 40, 1873–1882. 29093334 10.1248/bpb.b17-00321

[ece37665-bib-0107] Sunthonkun, P. , Palajai, R. , Somboon, P. , Suan, C. L. , Ungsurangsri, M. , & Soontorngun, N. (2019). Life‐span extension by pigmented rice bran in the model yeast *Saccharomyces cerevisiae* . Scientific Reports, 9, 1–16. 10.1038/s41598-019-54448-9 31792269 PMC6888876

[ece37665-bib-0108] Sutphin, G. L. , Bishop, E. , Yanos, M. E. , Moller, R. M. , & Kaeberlein, M. (2012). Caffeine extends life span, improves healthspan, and delays age‐associated pathology in *Caenorhabditis elegans* . Longevity & Healthspan, 1, 9. 10.1186/2046-2395-1-9 24764514 PMC3922918

[ece37665-bib-0109] Swanson, J. , Lancaster, M. , Anderson, J. , Crandell, M. , Haramis, L. , Grimstad, P. , & Kitron, U. (2000). Overwintering and establishment of *Aedes albopictus* (Diptera: Culicidae) in an urban La Crosse virus enzootic site in Illinois. Journal of Medical Entomology, 37, 454–460. 15535592 10.1093/jmedent/37.3.454

[ece37665-bib-0110] Thomson, J. D. , Draguleasa, M. A. , & Tan, M. G. (2015). Flowers with caffeinated nectar receive more pollination. Arthropod‐Plant Interactions, 9, 1–7. 10.1007/s11829-014-9350-z

[ece37665-bib-0111] Tower, J. (2011). Heat shock proteins and *Drosophila* aging. Experimental Gerontology, 46, 355–362. 10.1016/j.exger.2010.09.002 20840862 PMC3018744

[ece37665-bib-0112] Tu, C. P. D. , & Akgül, B. (2005). *Drosophila* glutathione S‐transferases. Methods in Enzymology, 401, 204–226. 16399388 10.1016/S0076-6879(05)01013-X

[ece37665-bib-0113] Valenzano, D. R. , Terzibasi, E. , Genade, T. , Cattaneo, A. , Domenici, L. , & Cellerino, A. (2006). Resveratrol prolongs lifespan and retards the onset of age‐related markers in a short‐lived vertebrate. Current Biology, 16, 296–300. 10.1016/j.cub.2005.12.038 16461283

[ece37665-bib-0114] Vontas, J. , Blass, C. , Koutsos, A. C. , David, J.‐P. , Kafatos, F. C. , Louis, C. , Hemingway, J. , Christophides, G. K. , & Ranson, H. (2005). Gene expression in insecticide resistant and susceptible *Anopheles gambiae* strains constitutively or after insecticide exposure. Insect Molecular Biology, 14, 509–521. 10.1111/j.1365-2583.2005.00582.x 16164607

[ece37665-bib-0115] Wright, G. A. , Baker, D. D. , Palmer, M. J. , Stabler, D. , Mustard, J. A. , Power, E. F. , Borland, A. M. , & Stevenson, P. C. (2013). Caffeine in floral nectar enhances a pollinator's memory of reward. Science, 339, 1202–1204. 10.1126/science.1228806 23471406 PMC4521368

[ece37665-bib-0116] Young, M. D. , Wakefield, M. J. , Smyth, G. K. , & Oshlack, A. (2010). Gene ontology analysis for RNA‐seq: Accounting for selection bias. Genome Biology, 11, R14. 10.1186/gb-2010-11-2-r14 20132535 PMC2872874

[ece37665-bib-0117] Yue, Y. , Shen, P. , Xu, Y. , & Park, Y. (2019). *p* ‐Coumaric acid improves oxidative and osmosis stress responses in *Caenorhabditis elegans* . Journal of the Science of Food and Agriculture, 99, 1190–1197. 30047165 10.1002/jsfa.9288

[ece37665-bib-0118] Zhang, H. , & Tsao, R. (2016). Dietary polyphenols, oxidative stress and antioxidant and anti‐inflammatory effects. Current Opinion in Food Science, 8, 33–42. 10.1016/j.cofs.2016.02.002

[ece37665-bib-0119] Zhang, Y. , Unnikrishnan, A. , Deepa, S. S. , Liu, Y. , Li, Y. , Ikeno, Y. , Sosnowska, D. , Van Remmen, H. , & Richardson, A. (2017). A new role for oxidative stress in aging: The accelerated aging phenotype in Sod1^−/−^ mice is correlated to increased cellular senescence. Redox Biology, 11, 30–37. 10.1016/j.redox.2016.10.014 27846439 PMC5109248

