# Supplementary figures and images for: Specific phytochemicals in floral nectar up‐regulate genes involved in longevity regulation and xenobiotic metabolism, extending mosquito life span
Source: Ecol Evol. 2021 May 25;11(12):8363–80. doi: 10.1002/ece3.7665 (PMC8216986; doi:10.1002/ece3.7665)

Color Key

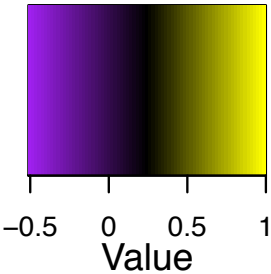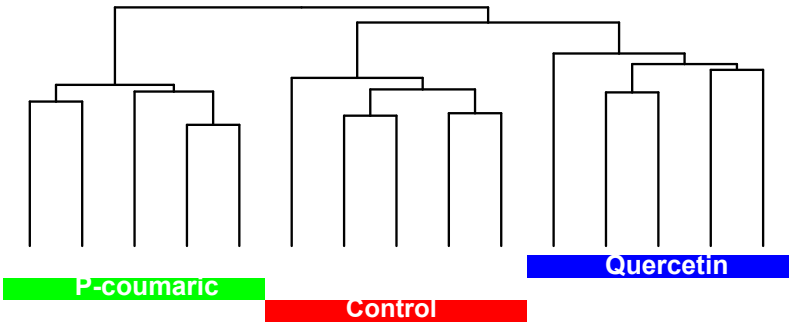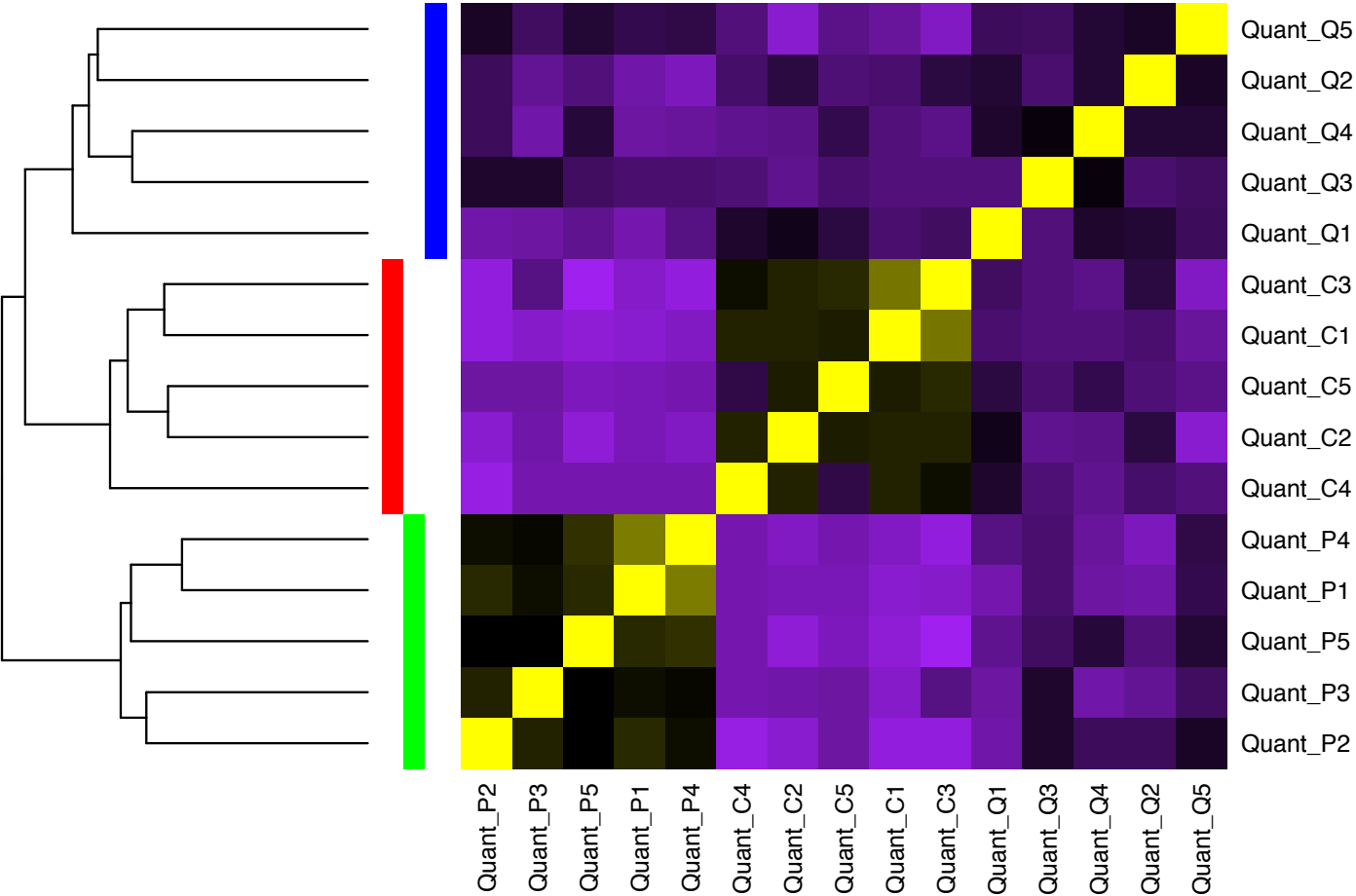

Supplement: Supplementary file 1 — Fig S1 [file ECE3-11-8363-s001.pdf]

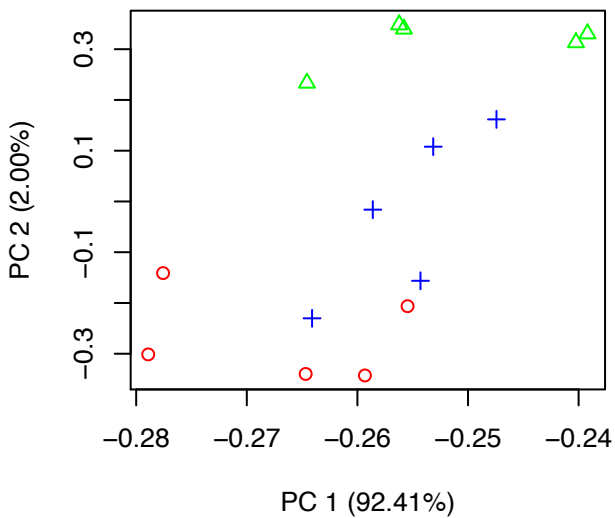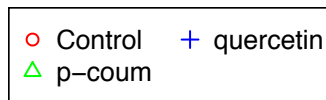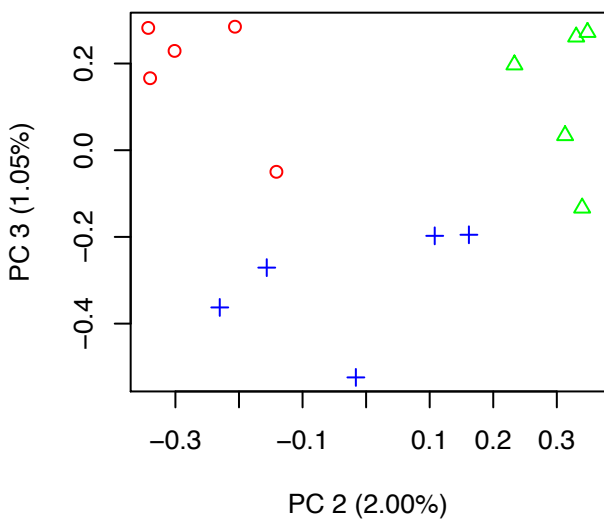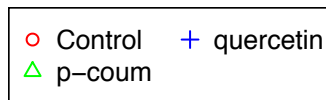

Supplement: Supplementary file 2 — Fig S2 [file ECE3-11-8363-s004.pdf]

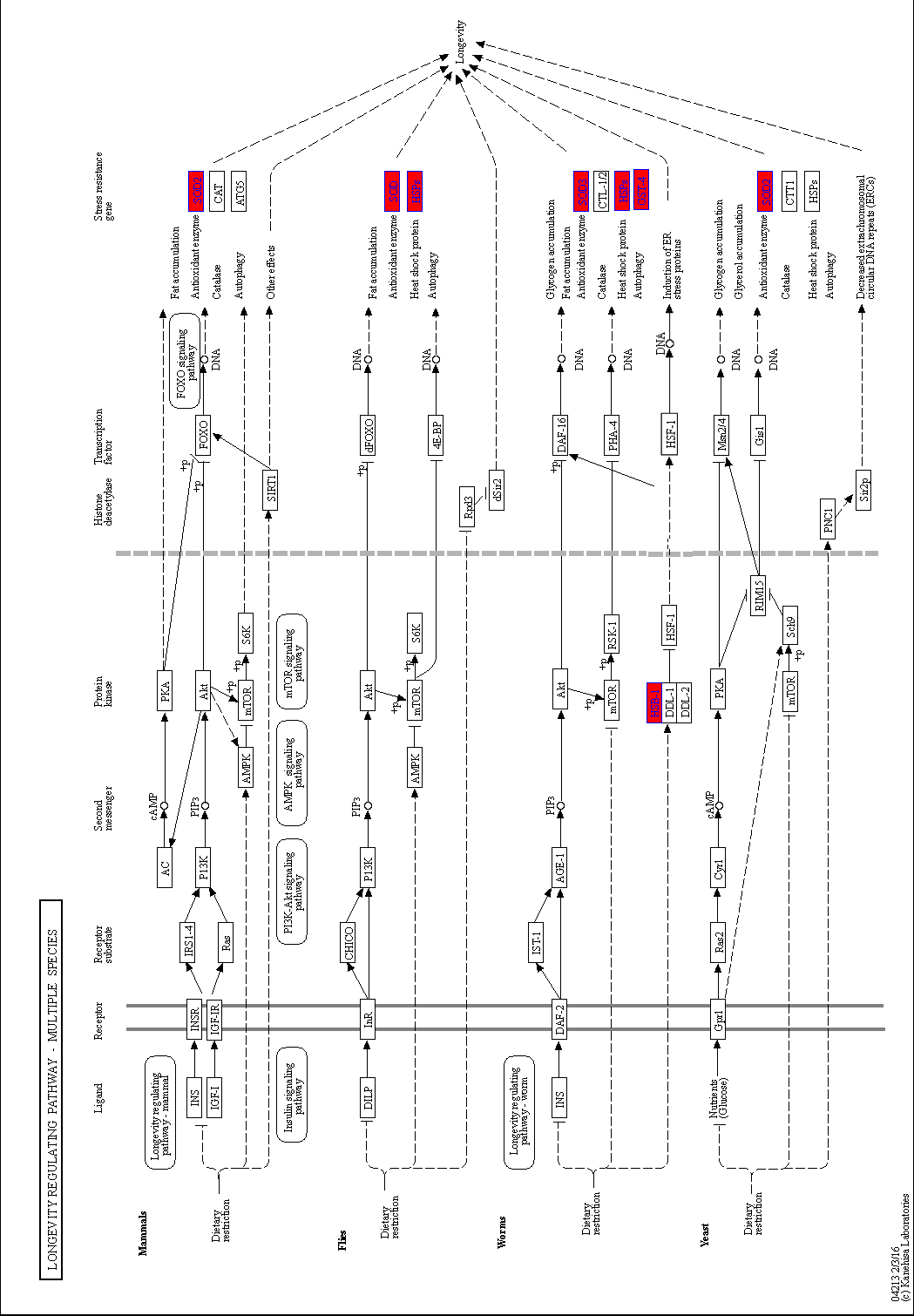

Supplement: Supplementary file 3 — Fig S3 [file ECE3-11-8363-s003.png]
