# Supplementary material for: Specific phytochemicals in floral nectar up‐regulate genes involved in longevity regulation and xenobiotic metabolism, extending mosquito life span
Source: Ecol Evol. 2021 May 25;11(12):8363–80. doi: 10.1002/ece3.7665 (PMC8216986; doi:10.1002/ece3.7665)
Supplement: Supplementary file 5 — Supplementary Material [file ECE3-11-8363-s002.docx]

**Figure S.1**: A heatmap showing the pairwise *Pearson* correlation of median-centered relative expression of transcript per million (TPM) values between samples for female *Ae. albopictus* consuming sucrose diets containing *p*-coumaric acid or quercetin and control. The dendrogram represent “relative similarity” as calculated by the PtR function in Trinity. Heatmaps were generated using built-in pheatmap function in R (v3.6.2) as implemented in Trinity.

**Figure S.2**: Principal component analysis of sample replicates for female *Ae. albopictus*consuming sucrose diets containing *p*-coumaric acid or quercetin and control. Principal components were calculated on the gene expression matrix using the “prcomp” function from R(v3.6.2), which uses singular value decomposition.

**Figure S.3**: Longevity-regulating pathway for genes upregulated with *p*-coumaric acid (adapted from KEGG pathways: hsa04213 and hsa04212). Genes upregulated in females *Ae. albopictus*consuming sucrose diet containing *p*-coumaric acid appear in red. Bold vertical lines are the cell membrane and dashed vertical line represents the nuclear membrane. Arrows connect genes/enzymes (in rectangles) in the pathway.
